# Population-based rare variant detection via pooled exome or custom hybridization capture with or without individual indexing

**DOI:** 10.1186/1471-2164-13-683

**Published:** 2012-12-06

**Authors:** Enrique Ramos, Benjamin T Levinson, Sara Chasnoff, Andrew Hughes, Andrew L Young, Katherine Thornton, Allie Li, Francesco LM Vallania, Michael Province, Todd E Druley

**Affiliations:** 1 Center for Genome Sciences and Systems Biology, Washington University School of Medicine, 660 S. Euclid Ave., St. Louis, MO, 63108, USA; 2 Department of Internal Medicine, Division of Cardiology, Washington University School of Medicine, 660 S. Euclid Ave., St. Louis, MO, 63108, USA; 3 Department of Pediatrics, Division of Hematology and Oncology, Washington University School of Medicine, 660 S. Euclid Ave., St. Louis, MO, 63108, USA; 4 Present address: Center for Genome Sciences and Systems Biology, Washington University School of Medicine, 4444 Forest Park Avenue, Box 8510, St. Louis, MO, 63108, U.S.A

**Keywords:** Rare variants, Genomics, Exome, Hybridization capture, Multiplexed capture, Indexed capture, SPLINTER

## Abstract

**Background:**

Rare genetic variation in the human population is a major source of pathophysiological variability and has been implicated in a host of complex phenotypes and diseases. Finding disease-related genes harboring disparate functional rare variants requires sequencing of many individuals across many genomic regions and comparing against unaffected cohorts. However, despite persistent declines in sequencing costs, population-based rare variant detection across large genomic target regions remains cost prohibitive for most investigators. In addition, DNA samples are often precious and hybridization methods typically require large amounts of input DNA. Pooled sample DNA sequencing is a cost and time-efficient strategy for surveying populations of individuals for rare variants. We set out to 1) create a scalable, multiplexing method for custom capture with or without individual DNA indexing that was amenable to low amounts of input DNA and 2) expand the functionality of the SPLINTER algorithm for calling substitutions, insertions and deletions across either candidate genes or the entire exome by integrating the variant calling algorithm with the dynamic programming aligner, Novoalign.

**Results:**

We report methodology for pooled hybridization capture with pre-enrichment, indexed multiplexing of up to 48 individuals or non-indexed pooled sequencing of up to 92 individuals with as little as 70 ng of DNA per person. Modified solid phase reversible immobilization bead purification strategies enable no sample transfers from sonication in 96-well plates through adapter ligation, resulting in 50% less library preparation reagent consumption. Custom Y-shaped adapters containing novel 7 base pair index sequences with a Hamming distance of ≥2 were directly ligated onto fragmented source DNA eliminating the need for PCR to incorporate indexes, and was followed by a custom blocking strategy using a single oligonucleotide regardless of index sequence. These results were obtained aligning raw reads against the entire genome using Novoalign followed by variant calling of non-indexed pools using SPLINTER or SAMtools for indexed samples. With these pipelines, we find sensitivity and specificity of 99.4% and 99.7% for pooled exome sequencing. Sensitivity, and to a lesser degree specificity, proved to be a function of coverage. For rare variants (≤2% minor allele frequency), we achieved sensitivity and specificity of ≥94.9% and ≥99.99% for custom capture of 2.5 Mb in multiplexed libraries of 22–48 individuals with only ≥5-fold coverage/chromosome, but these parameters improved to ≥98.7 and 100% with 20-fold coverage/chromosome.

**Conclusions:**

This highly scalable methodology enables accurate rare variant detection, with or without individual DNA sample indexing, while reducing the amount of required source DNA and total costs through less hybridization reagent consumption, multi-sample sonication in a standard PCR plate, multiplexed pre-enrichment pooling with a single hybridization and lesser sequencing coverage required to obtain high sensitivity.

## Background

Evidence that rare genetic variation modulates common, complex phenotypes and diseases continues to mount [1-4], which has spurred efforts to study larger affected cohorts in order to have adequate statistical power for causative rare variant identification. However, despite the ever-declining costs of sequencing, recent efforts to sequence entire exomes [5-7] or use genome-wide array data to inform candidate gene selection [1] result in a huge expansion of the number of candidate genes to be surveyed. Yet, sequencing a large number of candidate loci in thousands of individuals remains largely cost prohibitive in terms of capture costs, sequencing costs and bioinformatic capability for the standard investigator. To address this limitation, we previously pioneered efforts in cost-effective, population-based rare variant detection using pooled DNA sequencing methods and the pooled sequencing analysis tools, SNPseeker and SPLINTER [8,9]. While highly accurate and computationally efficient, these tools were designed for discreet target regions (e.g. <500 kb) and their aligners were inadequate for larger reference sequences.

In parallel, commercial capture-by-hybridization products have proven to be powerful tools for the custom enrichment of coding and/or other genomic regions of interest. These protocols are designed to hybridize complimentary oligonucleotide probes to the target regions of interest from a single DNA sample in a single incubation step. The efficacy of these methods to identify novel, deleterious monogenetic variants in Mendelian disorders has been reported in a variety of studies [4-7]. Newer products offer multiplexing of up to eight individuals in a single exome hybridization capture but require individual preparation of DNA libraries prior to hybridization, which increases DNA handling and reagent cost. Despite modest levels of available multiplexing, performing custom or exome hybridization capture on a large population of individuals followed by sequencing and data analysis remains a costly and time consuming proposition for most investigators.

To address these limitations, a variety of library preparation and multiplexing strategies, with and without individual indexing, have been put forth in the literature [10-18]. There are a variety of things to consider when selecting a pooled hybridization method. We have considered these technical aspects when designing and comparing our method: is indexing used or anonymous pooling; how large of a pool can be created; if indexing is used, is possible cross-contamination of indexes reported and quantified; are duplicate reads accounted for in the data analysis; what percentage of raw data is on or near-target; how large of a capture is performed; how uniform is the resultant data for each individual within the pooled or multiplexed sample; and, finally, how accurate are the results. Cost is certainly an important parameter for consideration, but is difficult to quantify given the rapidly changing environment and access to certain platforms at different institutions. While not all of these studies report their findings in each of these categories, we find that our strategy compares favorably or surpasses reported findings in many instances (detailed further in the Discussion), yet remains cost-effective and practical for smaller labs requiring manual sample preparation.

Obtaining accurate results from pools without individual indexing, even modestly sized pools of 20–50 individuals, can be quite challenging [10,11]. We have previously shown that the SPLINTER algorithm is highly sensitive for rare variant detection in pools of up to 500 individuals [9]. Previously, SPLINTER had only been used with PCR-amplified target sequences of modest total size. Here, we have expanded the capability of SPLINTER to larger target sequences acquired through solution hybridization capture without compromising its accuracy in a small pool of five exomes and in a larger pool of 92 individuals across 2.5 Mb. Our indexing strategy is compatible with low amounts of input DNA, less than 100 ng, which is often a limiting resource in large populations of DNA samples. This is primarily possible by eliminating all DNA sample transfers, including post-sonication, prior to hybridization and integrating a size-selection solid phase reversible immobilization (SPRI) bead cleanup strategy similar to that described by Fisher [19]. We employ a series of unique indexes that are effectively blocked using a single, novel blocking oligonucleotide incorporating deoxyinosine, rather than perfectly complimentary or degenerate sequences. This reduces cost and library complexity. We validated our methodology by comparing sequencing results to individual genotyping data and find an excellent concordance between pooled custom or exome hybridization capture results for common and rare variant detection. Overall, we believe this methodology provides a robust, scalable, accurate option for rare variant detection in complex phenotypes.

## Results and discussion

### Individual exome analysis

Initial reports of SureSelect [20] exome sequencing analysis used the program MAQ [21] to align short reads to the human genome [5,7]. Subsequent steps involved calling substitutions with MAQ and then using Cross_match [22] for INDEL calling. However, we have previously demonstrated that MAQ is inadequate for the analysis of pooled DNA samples [9], and wanted to use the same alignment tool for both the individual and pooled sample to minimize differences in variant calling due to variable alignments. Our first experiment was to compare Affymetrix 6.0 genome-wide array (GWA), individual exome and pooled exome sequencing from the same five individuals (Additional file 1: Table S1, rows 1–5). Because the aligner included in the SPLINTER software package was not designed for large reference sequences, we elected to use Novoalign for sequence alignment, which performs gapped alignments enabling simultaneous calling of substitutions and INDELs. We then used SAMtools [23] for variant calling. We called all variants with ≥5-fold coverage/chromosome (≥10-fold/base position) to demonstrate a high degree of accuracy at low coverage thresholds, which facilitates costs savings through reduced sequencing. There was an average of 16,040 total variants called/person (range 15,824-16,186) and an average of 1,194 novel variants per person (range 1,073-1,284). Additional file 1: Table S2 lists sequencing metrics for each individual.

To validate our individual and pooled sequencing results, we compared common positions between the exome sequencing and the GWA, as well as individual genotyping by Sequenom MassArray [24] for 127 positions, including six novel variants, not on the GWA and falling within genes of potential biological relevance for the phenotype under investigation within these individuals. Additional file 1: Figure S2A shows a highly reproducible average sensitivity across all five individuals of 96.9% (range 96.3 – 97.3%). Similarly, Additional file 1: Figure S2B shows a highly reproducible average specificity of 99.8% (range 99.7 – 99.8). Additional file 1: Table S3 lists results from individual genotyping compared to individual and pooled exome sequencing. We found that 95.3% were identical, including all novel variants, between individual exome sequence analysis and genotyping. These data demonstrate that our bioinformatic pipeline is not only computationally efficient, but also accurate even with as little as 5-fold coverage/chromosome.

### Pooled exome analysis

**Figure 1:**
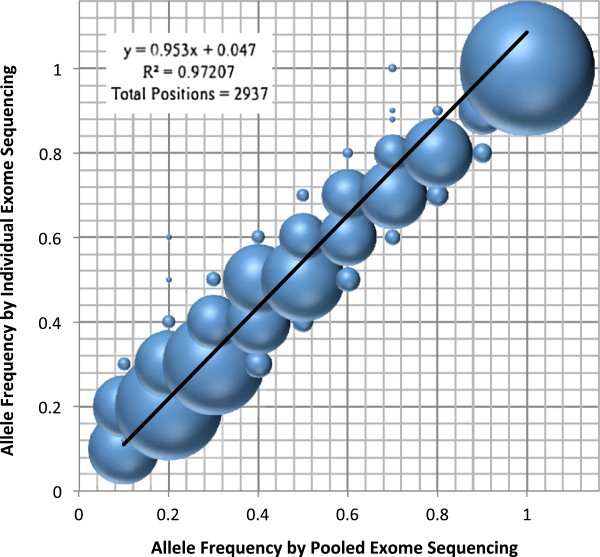
**Allele frequency by individual versus pooled exome sequencing.** Correlation plot comparing a total of 2,937 positions found in the Agilent SureSelect hybridization exome capture and Affymetrix 6.0 array between individual versus pooled exomes. Difference in size between the spheres represents the relative number of variant positions with the same minor allele frequency.

After establishing the accuracy of the single exome analysis, we focused on adapting the Novoalign alignment pipeline for integration with SPLINTER. To test this, we prepared a pool of normalized DNA from the same five individuals previously analyzed separately (pool size of 10 alleles). Systematic sequencing platform errors were modeled and subtracted from raw data as previously published [9], Additional file 1: Figure S1]. Additional file 1: Figure S2A shows that our pooled sequencing sensitivity slightly outperformed our individual exome sensitivity, demonstrating an accuracy of 99.4%. This improvement is likely due to the fact that we only considered called variants from pooled sequencing with ≥20-fold coverage based on prior results [9], supporting the idea that we could improve our individual exome variant calling sensitivity by restricting to positions with higher coverage thresholds. Figure 1 shows the correlation for substitution minor allele frequencies (MAF) between the individual and pooled sequencing to be *R*^2^ = 0.97, suggesting uniform capture and representation of the minor alleles in the pooled sample. Positions deviating from the idealized correlation are shown to be deficient in sequencing coverage in either the single or pooled sequencing (Additional file 1: Figure S3). Additional file 1: Figure S2B shows the specificity of the pooled hybridization capture to be 99.7%. Considering the non-array positions individually genotyped by Sequenom (Additional file 1: Table S3), we find a correlation of *R*^2^ = 0.81 between SPLINTER and either SAMtools or Sequenom. The lower correlation is due to seven no-calls by SPLINTER. For SPLINTER to call a variant, the complimentary base change must be noted on each strand to a degree that passes the defined P-value cutoff. In these cases, one strand failed to achieve adequate sequencing that surpasses the P-value threshold, thus leaving these positions uncalled.

### Pooled custom hybridization capture

**Figure 2:**
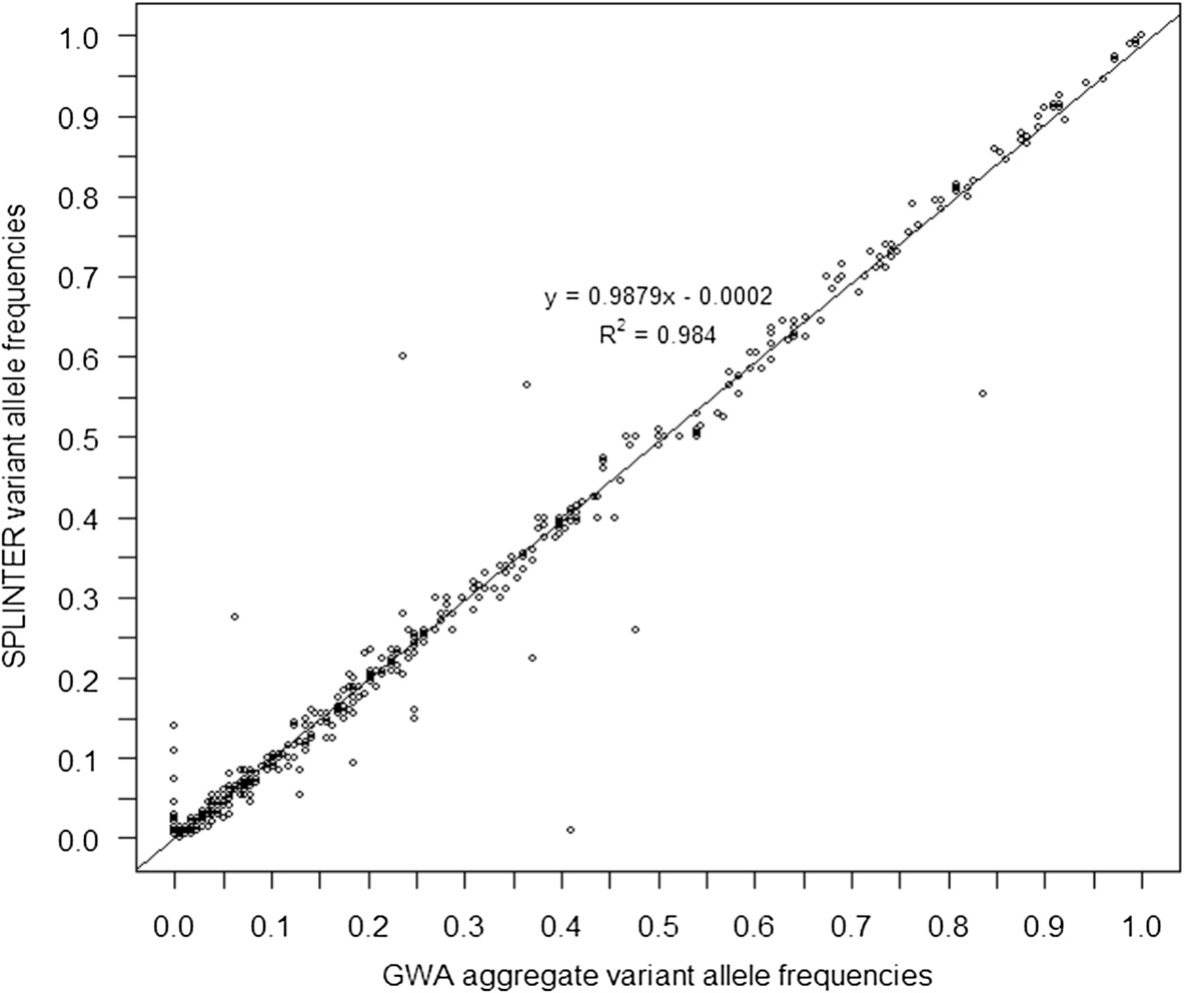
**Pooled capture SNV minor allele frequency correlation.** Correlation plot comparing a total of 499 positions that overlapped between the custom hybridization targeted regions and the Illumina Omni-2.5-8 genome wide SNV array with at least one variant called by the array or pooled analysis. Of these 499 positions, 477 (95.6%) had at least one variant allele call by both SPLINTER and the array, 20 were called as SNVs by SPLINTER but not by the array, and 2 were called as SNVs by the array but not by SPLINTER.

For this experiment, we analyzed non-indexed pooled sequencing results of 464 candidate genes (Additional file 1: Table S4) from 92 individuals enrolled in the Long Life Family Study (LLFS, see Methods for details). Each individual had Illumina HumanOmni 2.5-8 Beadchip GWA data available for comparison. Using filtering criteria listed in Methods under “Pooled sequencing analysis of 92 individuals at 464 genes”, we identified 875 substitutions for comparison. Of these, 499 were called polymorphic at some frequency by SPLINTER or the GWA, yielding a concordance of *R*^2^ = 0.984 (Figure 2). There were 116 rare substitutions within this dataset (MAF <5%) and 104 (89.7%) were called within 1% of the array-based MAF by SPLINTER, 2 were not called and the remaining 10 were called within 2% of the array-based MAF. At least some of the discrepancies were likely due to GWA errors. When considering only the 396 substitutions called homozygous wild type by GWA, we find a specificity of 94.9%. However, if we exclude positions from consideration that have an INDEL within 15 bp called by sequencing in at least one individual, 25 positions are excluded and our specificity improves to 97.8%. This suggests false negative array errors due to nearby INDELs that adversely affect probe binding.

For INDEL analysis in this experiment, we aggregated the INDEL calls from the individual sequencing of all 92 individuals and compared against SPLINTER’s calls. When restricting our analysis to 1 bp INDELs, SPLINTER identified 90% (99 of 110) with a concordance of *R*^2^ = 0.73 (Additional file 1: Figure S4). For rare INDEL calling (MAF <5%), SPLINTER detected 83.3% (45 of 54). While these results are good overall, the dropoff in concordance from substitution detection to that of INDELs further highlights the difficulty of accurate INDEL detection.

Thus, we have expanded the prior capability of the SPLINTER algorithm for use with any size target sequence. SPLINTER was previously shown to outperform other algorithms, such as CRISP [18], for rare variant and INDEL detection in anonymous pools of up to 500 individuals [9], but the aligner was not efficient for survey of large reference sequences. Recent reports of anonymous pooling from smaller pools of 2–50 individuals [10,11,18] use hybridization capture on smaller capture targets (up to 1.6 Mb). Both studies removed duplicate reads prior to alignment and report 48-55% of data on target (compared to our 42-47%). However, Harakalova failed to validate any called variant predicted at less than 25% MAF in pools of 20 [11]. Day-Williams found that solution hybridization capture outperformed PCR or array-based hybridization methods and report a sensitivity and specificity of 98.2% and 97.0% for a smaller pool of 20 individuals using MAQ v0.1.7 for alignment and Syzygy for SNV calling with no analysis of INDELs. Bansal and colleagues surveyed 594 kb in pools of 20 and report a sensitivity of 93% at 30-fold coverage for substitutions and a positive predictive value of 87% for INDELs compared to a false positive rate of 0.9%. In comparison, we find a sensitivity of >96% at 20-fold coverage in a pool of four captures of 22–24 each.

### Indexed custom hybridization capture

While pooling samples without indexing is cost-effective for surveying sequence variation between populations, it’s obviously advantageous to directly link a called variant to a particular individual within the multiplex. A variety of studies have introduced barcodes or indexing into multiplexes ranging in size from 5–96 individuals for target sizes of 0.14-3.3 Mb [12-18]. These reports start with anywhere from 0.2-3 μg of DNA per individual. Rohland and Reich created libraries with as little as 200 ng of DNA/person but report 50% sample loss from their blunt end adapter ligation strategy and therefore recommend using ≥500 ng of DNA/pp [16]. We have also adopted a blunt-end adapter ligation strategy incorporating Y-shaped adapters and have not seen significant sample loss, which has enabled comparable outcomes from libraries starting with 70 ng/pp up through 350 ng/pp. In addition, we present a novel, simplified adapter blocking strategy using deoxyinosines along with a custom set of 96 indexes (Additional file 1: Table S5).

In this experiment, we analyzed sequencing data from the same 464 candidate genes (Additional file 1: Table S4) in 280 LLFS individuals and incorporated individual DNA indexing prior to pre-hybridization multiplexing. To test pre-enrichment multiplexing of various sizes, we created nine separate multiplexes of 22 (n = 1), 23 (n = 2), 24 (n = 1), 30 (n = 2), 32 (n = 1) and 48 (n = 2) and using 70–350 ng DNA/pp. Additional file 1: Table S1 lists how the multiplexes were then combined for three separate lanes of HiSeq 2000 sequencing with 92–96 individuals in each lane. We first studied the single lane of 92 individuals from four multiplexes of 22–24 individuals each (rows 7–10), and found that >96% of raw data contained an identifiable index and that >97% of the individuals (2 samples failed) were within a 2.3-fold difference in raw read counts (Additional file 1: Figure S5). Also, individuals did not cluster predominantly within distinct captures (compare the distribution of symbols within each dataset in Additional file 1: Figure S5), suggesting that the library preparation and hybridization was highly uniform and did not introduce bias in the sequencing output. After excluding the two failed samples, aligning unique reads and removing PCR duplicates, 81.9% (±1.8) of total reads per person were aligned in a trend that closely followed the number of raw reads per sample (Additional file 1: Figure S5, cyan symbols). We observed 13.4% (±1.9) PCR duplication (Additional file 1: Figure S6, red symbols). After removing these duplicates, 41.8% (±1.1) of the aligned bases were on or within 250 bp (“on or near target”) of our targeted intervals and 33.4% (±0.9) were directly on targeted regions (Additional file 1: Figure S6, blue and green symbols respectively), demonstrating that the percent duplication and capture efficiency were uniform between all samples and the four independent multiplexes. In addition, we demonstrated that our candidate regions were enriched uniformly across the four multiplexes, showing an average pairwise *R*^2^ = 0.936 (Additional file 1: Figure S7). Thus, our method performs uniformly with respect to both aggregate metrics of an entire capture as well as bait-to-bait capture efficiency.

As discussed by Rohland and Reich [16], many capture methodology papers do not report the number of duplicate reads observed nor specify if duplicate reads are removed prior to alignment [12-14,17,18] and only report the percentage of their reads that align on or near target, not directly on target, which may inflate the amount of usable data. Rohland reports 24% duplicate reads, and we find a comparable percentage of 14-32% duplicate reads in our various multiplexes. While our percentage of on/near target reads is comparable to the 18-55% on/near target reads reported [12-15,17,18], these numbers may be inflated by the inclusion of duplicates. In our initial multiplexed samples, we find an average of 81.9% of our reads remaining after aligning and removing duplicates, and an average of 33.4% of the remaining bases aligning to our targeted regions. Thus, approximately 27.4% of our raw data aligns to our targeted regions in our processed aligned files, yielding an average fold enrichment of 413 (±10.8). Overall, we achieved an average mean target coverage of (30.43 ± 4.54) when targeting 2.5 Mb with one lane of sequencing and yielding ~130 million reads. While within range of prior data, we expected to obtain a higher on-target percentage. The most obvious reason for this seemed to be our modified adapter blocking strategy allowing daisy-chaining, but could also be due to limitations in oligonucleotide hybridization efficiency either through non-specific hybridization to non-target genomic regions or because early versions of Agilent SureSelect custom captures did not incorporate “bait boosting”, which calibrates the relative amount of bait sequences to normalize the expected binding efficiency to each target. While we could not alter the baits within our capture set, we did improve our blocking strategy by incorporating 1 μL of the Illumina PE 1.0 post-hybridization PCR primer sequence (Additional file 1: Table S5, the longer oligonucleotide listed under “Post-Hybridization PCR primers”). This step improved our average on-target percentage to >70% over our next 18 multiplexed libraries (data not shown), which is comparable to the 79% on/near target reads using a shortened adapter strategy published by Rohland [16].

Analysis of the data from the lane with two 48-individual multiplexes (Additional file 1: Table S1, rows 14–15) found very similar results for what we observed from multiplexes of 22–24 with respect to the percent of raw reads with an identifiable index, the percentage of aligned data per person, and the uniformity of data between individuals and between captures (Additional file 1: Figure S5, black symbols). This experiment showed a higher fold enrichment of 466 (±17.2) with 31.6% (±1.5) PCR duplication (Additional file 1: Figure S8, red symbols). After removing these duplicates, our on/near-target percentage improved to 47.2% (±1.4) and 37.7% (±1.39) were directly on targeted regions (Additional file 1: Figure S8, blue and green symbols respectively). We attribute the higher PCR duplication rate in the multiplexes of 48 individuals over the multiplex of 22–24 individuals to the fact that we have reduced the library complexity through two mechanisms. First, we only pooled 35 ng of pre-hybridization capture DNA per individual instead of 50 ng as done with the multiplexes of 22–24, which would result in a lesser amount of unique DNA fragments available for the post-adapter ligation enrichment PCR. Second, with 48 individuals pooled for a single bait set, there is approximately half as much DNA selected per individual as for captures with 22–24 individuals pooled. Thus, with similar overall read counts per person, we would expect to see a greater number of PCR duplicates as a result.

**Figure 3:**
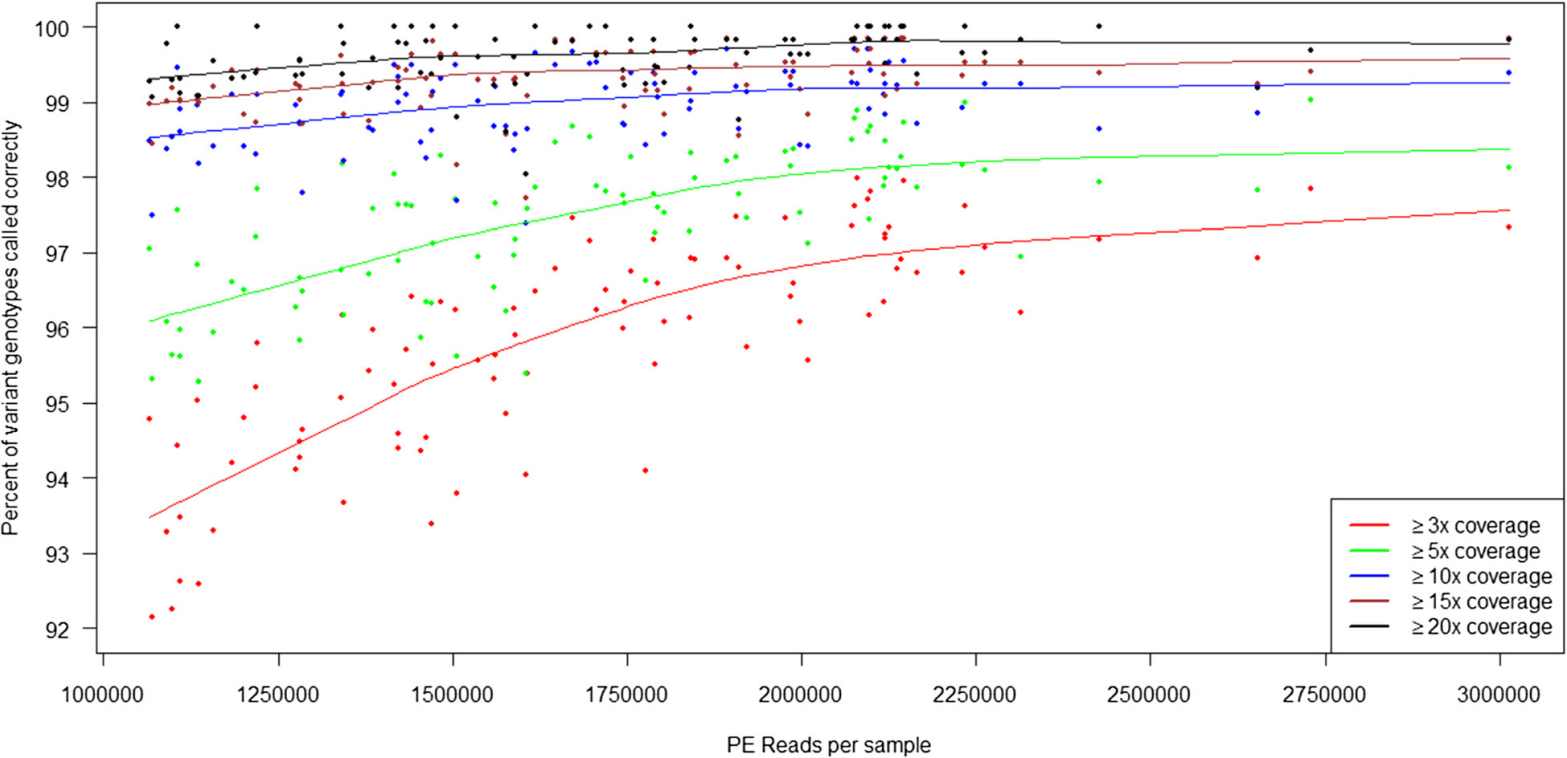
**Sensitivity as a function of total paired-end read counts at different coverage thresholds for all variants in the sets of 48 multiplexed samples.** This graph shows how the sensitivity increases per the number of reads for a given sample with a shallow increase at 5-fold coverage after a sample reaches 1.8 million reads. Sensitivity is defined as the percentage of heterozygous or homozygous variant genotypes as seen by the array called correctly as heterozygous or homozygous by sequencing. Red symbols: ≥3x coverage. Green symbols: ≥5x coverage. Blue symbols: ≥10x coverage. Brown symbols: ≥15x coverage. Black symbols: ≥20x coverage. Trend lines were generated using the “lowess” function in R with default parameters. The sensitivity appears to plateau at around 1.8 million reads, which is prior to excluding duplicate reads and reads that align off-target. PE = paired-end.

**Figure 4:**
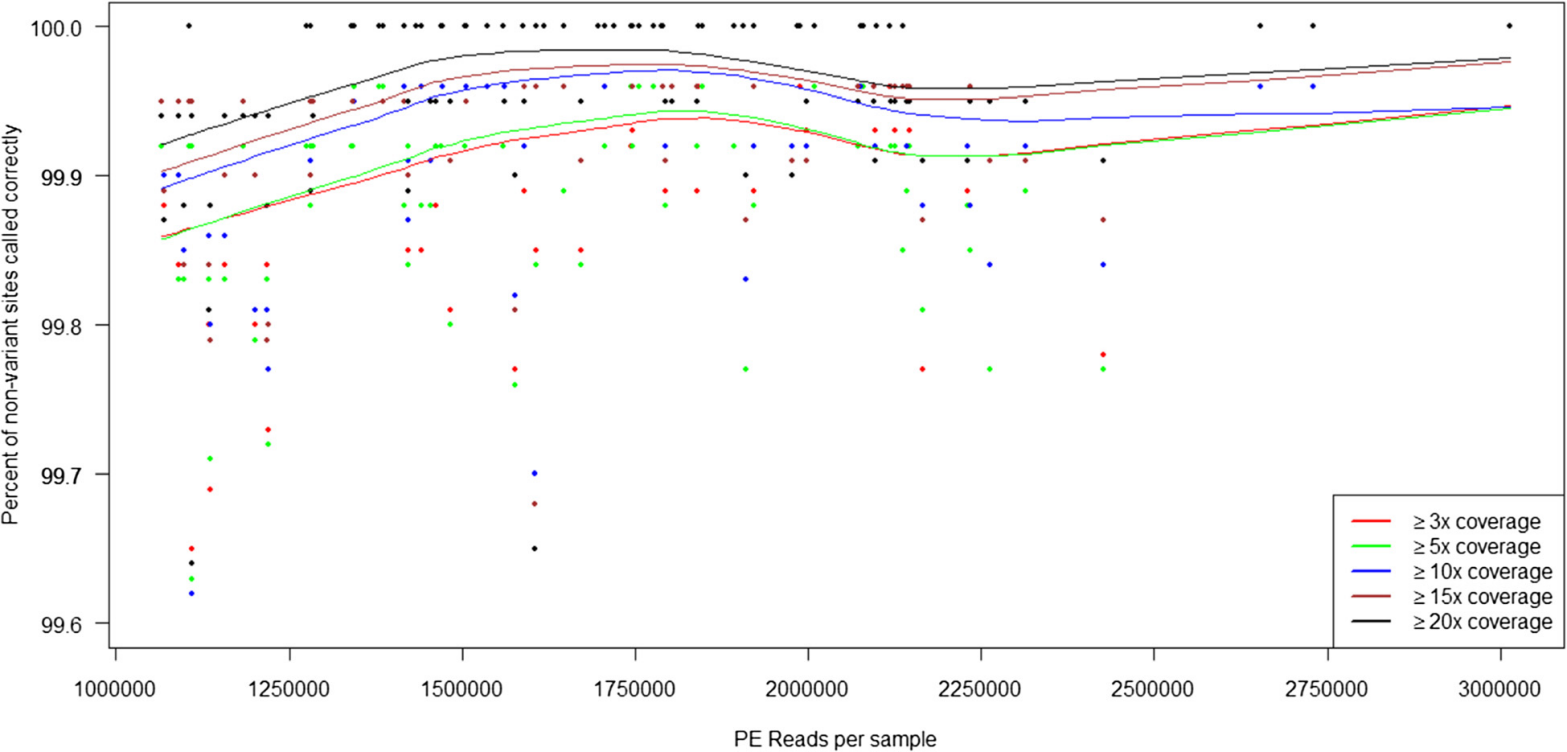
**Specificity as a function of total paired-end read counts at different coverage thresholds for the sets of 48 multiplexed samples.** Coverage has little impact on specificity, which starts at ≥99.8% for most individuals with >1.25 million reads. Specificity is defined as the percentage of homozygous wild type genotypes as seen by the array called correctly as homozygous wild type by sequencing. Red symbols: ≥3x coverage. Green symbols: ≥5x coverage. Blue symbols: ≥10x coverage. Brown symbols: ≥15x coverage. Black symbols: ≥20x coverage. Trend lines were generated using the “lowess” function in R with default parameters. The dip in specificity at around 2.25 million reads is likely due to noise as a single extra false positive can cause the observed shift downward. PE = paired-end.

As there were over 4,000 overlapping positions between the Omni 2.5-8 array and our targeted regions, we used these validated positions to accurately assess our sensitivity and specificity at various coverage thresholds. Table 1 shows sensitivity, specificity, and coverage metrics for the custom multiplexes at different MAF thresholds. As expected, sensitivity is a function of coverage, starting at >95% with >3-fold coverage for common variants and improving to 100% with >20-fold coverage for rare variants (Table 1 and Figure 3). Specificity was consistently ≥99.8% and hardly impacted by coverage regardless of MAF, confirming that our method does not systematically introduce false positives (Table 1 and Figure 4). For rare variants with ≤2% MAF in this experiment, we find a sensitivity of 92.9-99.6% and 94.7-100% with coverage ranging from ≥3-20-fold for variants with 2% MAF and 0.5% MAF, respectively (Table 1). Specificity was ≥99.99% at all coverage levels for either MAF. These results indicate that allele dropout is not occurring with our method.

**Table 1:** Comparing sensitivity and specificity to coverage achieved for three sets of multiplexes

			**All allele frequencies**					**≤5% MAF**					**2% MAF(4 alleles in pool)**			**0.5% MAF(1 allele in pool)**		
**Size Multiplex**	**Coverage**	**Percent of Bases, average (SD)**	**Homozygous wild type sites**	**Heterozygous sites**	**Homozygous variant sites**	**Sensitivity average (SD)**	**Specificity, average (SD)**	**Homozygous wild-type sites**	**Heterozygous sites**	**Homozygous variant sites**	**Sensitivity (avg)**	**Specificity (avg)**	**Heterozygous sites**	**Sensitivity (avg)**	**Specificity (avg)**	**Heterozygous sites**	**Sensitivity (avg)**	**Specificity (avg)**
22-24	≥3	84.4(1.29)	229483	39100	24855	95.02(1.42)	99.82(0.14)	172205	2057	20	92.49	99.98	656	91.6	99.98	127	88.3	99.99
	≥5	84.6(1.75)	221031	37332	23810	96.75(1.23)	99.82(0.14)	166050	1961	19	95.30	99.98	624	94.9	99.99	119	90.2	99.99
	≥10	75.0(2.65)	201268	33626	21451	98.22(0.98)	99.86(0.13)	151395	1768	17	97.42	99.98	563	96.6	99.99	111	93.8	100
	≥15	67.1(3.57)	182332	30279	19510	98.60(0.93)	99.88(0.12)	137327	1579	17	98.50	99.98	507	98.0	99.99	100	96.0	100
	≥20	59.8(4.51)	163986	27202	17643	98.94(0.83)	99.89(0.12)	123572	1418	14	98.81	99.98	460	98.7	100	90	95.6	100
48	≥3	89.0(2.30)	239300	39218	26310	95.80(1.42)	99.90(0.08)	187077	2109	25	92.97	99.97	651	92.9	99.99	131	94.7	100
	≥5	84.1(3.14)	230685	37434	25232	96.43(0.95)	99.91(0.08)	179900	1997	25	95.95	99.97	619	95.8	99.99	125	96.8	100
	≥10	74.8(4.54)	209960	33448	22739	98.96(0.51)	99.94(0.07)	163128	1805	22	98.30	99.97	566	98.4	99.99	111	100	100
	≥15	67.1(5.79)	190652	30010	20491	99.33(0.42)	99.95(0.07)	147843	1630	19	98.85	99.97	517	99.0	99.99	100	100	100
	≥20	60.3(6.98)	172373	26937	18505	99.60(0.37)	99.96(0.06)	133259	1449	18	99.05	99.97	467	99.6	100	88	100	100
30-32	≥3	89.2(2.55)	245952	40732	26487	96.52(1.08)	99.97(0.03)	184498	1903	16	93.54	99.97	664	95.6	99.98	102	97.1	99.99
	≥5	84.4(3.49)	235796	38696	25251	98.23(0.69)	99.97(0.03)	177279	1809	16	96.33	99.97	644	97.1	99.99	102	97.1	100
	≥10	74.8(5.35)	212716	34440	22505	99.43(0.40)	99.97(0.03)	160352	1600	14	98.88	99.97	567	99.1	99.99	99	96.8	100
	≥15	66.8(7.25)	192642	30957	20272	99.80(0.19)	99.98(0.03)	145504	1403	12	99.58	99.98	497	99.4	100	82	97.6	100
	≥20	59.5(9.08)	173212	27775	18237	99.90(0.15)	99.98(0.03)	130959	1260	10	99.76	99.98	448	99.6	100	72	98.8	100

The percent of bases in the targeted intervals that reached the specified coverage threshold is listed. Homozygous wild type, heterozygous, and homozygous variant sites surveyed indicates the number of each of those positions as seen by the Illumina Omni 2.5-8 array that were used to determine sensitivity and specificity. Sensitivity is the percentage of heterozygous and homozygous variant sites correctly called heterozygous and homozygous variant, respectively. Specificity is the percentage of homozygous wild type sites called as homozygous wild type. For “All allele frequencies” these values were averaged among all non-excluded samples. For ≤5**%**, 2% and 0.5% minor allele frequencies, these values were determined from the cumulative metrics of all non-excluded samples at sites with 9 or fewer, 4 or fewer, or 1 variant allele in the entire pool, respectively.

Additional file 1: Figure S9 compares sequencing coverage from a single individual for all common GWA positions against all bases in our 2.5 Mb capture with 1.4 M paired-end reads. Once a base position surpasses 3-fold coverage, the representation of GWA positions versus any base position with similar coverage is approximately equivalent (as are the mean and median values). These results support using high-density GWA positions for validation of sequencing results and extrapolating to the entire dataset. We then analyzed our non-array variant calls from our multiplexes of 48 (Table 1, rows 14–15) for accuracy. Any variant found in the Exome Variant Server and not considered a likely false positive by their Support Vector Machine (SVM) [25] was considered. Of the 10,854 non-SVM, non-GWA variant calls made, 9,622 are in dbSNP135 (88.65%). This degree of overlap would not be observed if variants were being called by random chance.

We have also surveyed all variation in our multiplexes of 22–24 individuals (Additional file 1: Table S1, rows 7–10) to calculate the Ti:Tv ratio according to the work of DePristo [26] (for details see Additional file 1: Results under “Transition : Transversion (Ti/Tv) ratio analysis”). We find additional support that we are calling variants as expected, particularly those positions with ≥15-fold coverage.

Often, DNA samples are a precious resource, with very little material in which to conduct large scale genomic surveys. We have been able to use relatively little source DNA per person by eliminating all sample transfers prior to pooling, which is enabled by having SPRI beads present in solution from the beginning of the protocol. These beads can stay in place and do not appear to alter the quality of the library creation reactions. Our strategy is modified from prior reports by Rohland [16] and Fisher [19]. Many investigators do not have access to full automation of the hybridization capture library creation process. Fisher introduced exclusive bead cleanup for use with fully automated single exome library creation, without multiplexing. While results were highly uniform when employed with full automation, there was a large dropoff in the percentage and uniformity of captured bases when bead cleanup was performed manually [see ref. [19], Additional File 10. Rohland performed sonication in a 96-well PCR plate without beads present in solution (unlike us) and also optimized their bead cleanup for automation. However, as mentioned above, they report a loss of 50% of source DNA after adapter ligation. We have exclusively performed manual library preparations with multichannel pipettors and observe excellent recovery of source DNA, which is a cost effective and accurate alternative that is accessible to any lab.

We investigated whether issues such as bait GC content or strand bias adversely impacted our results. We observed a large dropoff in sequencing coverage with baits that were high in GC content and targets <180 bp (discussed further in Additional file 1: Figures S10a and 10b), and performed an analysis to centeracterize whether strand bias impacted our variant calling (Additional file 1: Figure S11). From these results, we believe that GC bias in hybridization efficiency can be mitigated through “bait boosting” (or designing baits with no more than 35% GC content) and strand bias did not adversely effected our results.

However, one serious potential drawback in integrating large scale indexing would be data contamination due to index switching, or the transfer of an index from one individual to the sequencing read of another individual. Unexpectedly, the multiplexes of 22–24 and 48 demonstrated superior specificity for rare variants rather than common variants, which seems counterintuitive, but is a consistent finding at all coverage thresholds (Table 1). To explore this result further, we considered the validated positions binned by the number of variant alleles at each position called by array genotyping in the entire pool of 96 captured in two multiplexes of 48 (Additional file: 1 Table S6). The percentage of reads with variant alleles in individuals who were homozygous wild type at these positions increases as a function of the frequency of the variant allele in each bin. This indicated to us that there was up to 9% of reads with an inappropriate index in our samples. We use a large excess of adapters to drive ligation of every possible template strand. We surmised that a large percentage of our misattributed indexes were due to an excess of unligated adapters present in the PCR enrichment, as shown by the bimodal peaks in the green tracing on Additional file 1: Figure S12 and schematically diagrammed in Additional file 1: Figure S13. We tested this by adding an additional bead purification step to remove to remove unligated adapters from our multiplexes of 30–32 individuals (Table 1, rows 11–13). We further analyzed variants with a MAF of ~50% (Additional file 1: Table S6) and see an equivalent specificity for common and rare variants with the inclusion of this step (Table 1, rows 11–13). In addition, the lesser degree of misattributed indexes in the multiplexes of 30–32 improves sensitivity for common and rare variants compared to the prior multiplexes. Our results suggest that index switching is a random and stochastic process resulting in approximately 3.4% of reads in the entire pool attributed to an incorrect index. We propose a mechanism for index switching in Additional file 1: Figure S13. None of the previously mentioned reports on multiplexed hybridization capture discuss this [12-18] which may further skew their findings, but one possible source of this observation is covered in detail by Kircher and colleagues [27] as “jumping PCR”. Kircher reports that over 0.7% of misattributed indexes are expected when pooling samples prior to PCR for multiplexed capture. If we subtract this source of error from our result, we are left with 2.64% of reads with an incorrect index (a 3.5-fold improvement from initial experiments). The source of this is unclear, but may be caused by further residual unligated indexes in our sample prior to PCR enrichment, a different rate of “jumping PCR” due to innate differences in polymerases used (Amplitaq Gold by Kircher vs. the TruSeq kit reagents), or perhaps erroneous array calls. Thus, while our percentage of incorrectly indexed reads was not as low as reported by the double indexing strategy of Kircher [27], the amount we did have did not appear to impact our accuracy for common or rare variant detection.

## Conclusions

The genomic analysis of rare variants in complex disease will continue to drive pathophysiological research and clinical questions. Sequencing many individuals at any number of candidate loci is required to obtain the statistical power necessary for detecting disease-related, rare functional genetic variants associated with complex phenotypes. As sequencing capacity continues to increase, the need for additional multiplexing is required to maximize the efficiency of the sequencing output. Pooling will also enable sequencing to move more readily into the diagnostic arena as a more cost effective method for screening multiple genes for rare functional variation. We report a cost-effective and fully scalable, pre-hybridization multiplexed in-solution capture method, amenable to <100 ng of input DNA per person due to removal of sample transfers during library preparation enabled by the constant presence of SPRI beads and resulting in a 50% reduction in the volume of library preparation reagent necessary. Sequencing analysis was performed by bioinformatic pipelines aligning raw data with Novoalign and calling variants from anonymous pools using SPLINTER or indexed samples using SAMtools. Both pipelines showed consistent, coverage-dependent high sensitivity ranging from 95-99% for substitutions with a dropoff in concordance for detecting INDELs. For rare variants (MAF <2%), we achieved a sensitivity and specificity of 99.4% and 99.7% for pooled exome analysis and ≥96.6% and ≥99.99%, respectively, for custom capture libraries of 22–48 individuals. While within the range of published reports, we found our on-target percentage to generally be in the 40-50% range, but has improved with the addition of another post-enrichment PCR primer to act as an additional blocker. Our blocking strategy utilizes deoxyinosines to span the novel 7 bp indexes rather than degenerate nucleotides to reduce the complexity of the DNA mixture. In our system, we found that optimal hybridization and sequencing representation occurred with baits having 25-35% GC content and targets of >240 bp. We also found no negative impact due to strand bias. However, one serious drawback to larger multiplexing of indexed samples is index switching leading to the attribution of variants to the incorrect individual. We have quantified the prevalence of this phenomenon and shown it to occur most frequently at wild-type or common variant positions simply due to the abundance of those reads in the sample. By incorporating an additional purification step during library preparation to remove excess unligated adapters, this effect can be mitigated. In sum, cost savings can be realized through the use of less pre-enrichment library preparation reagents, use of more standard plasticware for sonication, capturing up to 48 individuals with a single hybridization, multiplexing multiple libraries for sequencing, and the ability to call rare variants accurately with as little as 5-fold sequencing as a lower threshold (albeit sensitivity improves with higher coverage). While there are a variety of reports describing pooled sequencing or multiplexed solution hybridization capture [10-18], our method offers a highly uniform, reproducible workflow for smaller labs that may not have access to high-throughput robotics but still wish to quantify rare variation within specific populations.

## Methods

### Individual and pooled exome sequencing library preparation

DNA was isolated from buccal swabs collected from a nuclear family of five Caucasian individuals (four females and one male) in accordance with Washington University Human Research Protection Office protocol #10-0340. DNA was prepared for sequencing according to the protocol included with the Illumina Paired-End Sample Prep Kit (cat# PE-102-001; San Diego, CA). Additional file 1: Table S1 summarizes the amount of input DNA used for the individual and pooled exomes along with the sequencing platform and enrichment kit used. The five individual samples and the pooled sample then proceeded through each step of the library preparation protocol as described in the accompanying Agilent protocol.

### Exome enrichment

Exome enrichment from the prepared DNA libraries was performed using the Agilent (Santa Clara, CA) SureSelect Human All Exon Kit [20] for Illumina Paired-End Sequencing Library Prep targeting 38 Mb of the human genome. Hybridization, recovery and enrichment were done according to protocol version 1.0.1, October 2009 with no differences from the stated protocol for the single individual samples. For the pooled sample, two parallel hybridizations were performed and then combined prior to magnetic bead purification in order to optimize the amount of uniformly hybridized sample available for downstream reactions.

### Individual and pooled exome analysis

Raw sequence data in fastq format was aligned to the NCBI human genome build 36 (hg18) [28] using a purchased, multi-threading version of Novoalign [29]. Variant calling from the aligned output for the individual exomes was then performed using SAMtools [23] to manipulate alignments in SAM format and call substitutions as well as insertions and deletions (INDELs).

Raw sequence data in fastq format was aligned with Novoalign version 2.05. An alignment threshold of 200 was used (−t 200), with adapter stripping (−a AGATCGGAAGAGCG) and quality calibration enabled (−k). Reads with multiple alignments were discarded (−r none, –e 1) and output was in SAM format (−o SAM). Header lines were removed and the aligned data was then converted to BAM format with SAMtools “import” and sorted with SAMtools “sort”. Duplicate reads were then removed using Picard “MarkDuplicates”. The resulting file was sorted again and indexed with SAMtools “index”. Variants were called with the SAMtools version 0.1.18 mpileup command, using options -AB –ugf and bcftools “view” with settings -bvcg. Variants were filtered with vcfutils “varFilter” using default settings except retaining all variants with under 99999 reads.

For the pooled exome sample, the alignment was performed using Novoalign using a reference sequence for the 165,637 bait intervals from the exome capture plus 76 bp flanking bases. The alignment settings are as described above with the exception of output “Native” format instead of SAM format, and the aligned data was converted to a format consistent with the SPLINTER aligner output for SNV calling [30]. These modified files were used as input for variant calling using SPLINTER [9]. To minimize memory limitations during the variant calling process the human genome reference sequence was divided into 166 files with ~1,000 capture baits per file, each file was run as a separate job and parallelized one job per available CPU core. The settings for SNV variant calling using SPLINTER6r were as follows: 24 cycles incorporated, -1.3 p-value cutoff (log10 scale), using only unique reads, 2 edits (mismatches from reference) allowed, specifying a maximum of 10 alleles in the pool. Our prior reports on pooled sequencing relied on internal controls to model sequencing errors and establish a P-value that could be used to calibrate SPLINTER’s sensitivity and specificity for variant calling. The number of sequencing cycles to be incorporated was chosen as described by Vallania [9] based upon error rates identified in 392 base pairs in the *FABP1* gene (exon 3 plus 76 bp flanking sequence) with no called variants on the arrays or sequencing (Additional file 1: Figure S1). The model created from this negative control is used by SPLINTER to optimize specificity by subtracting systematic artifacts.

### Pooled sequencing and indexed custom library preparation

The entire protocol is detailed in the Supplemental Methods. The number of individuals multiplexed in each capture reaction and the multiple captures combined for sequencing within one lane of the Illumina HiSeq 2000 platform are detailed in Additional file 1: Table S1. Customized adapter, blocker, pre- and post-hybridization PCR amplification primers and all index sequences used are listed in Additional file 1: Table 5. Briefly, individual DNA samples were collected following informed consent by the Long Life Family Study (LLFS) [31]. For custom hybridization capture of 464 candidate genes thought to be relevant for extreme longevity (Additional file 1: Table 4), a bait set was created using the Agilent eArray online tool [32] for the Agilent SureSelect Custom DNA Capture [20] using 2X tiling frequency, a exon-centered layout strategy, and eliminating probes that overlap standard repeat masked regions by 20 or more bases. Of the 6,630 targeted intervals, 6,369 were covered by at least one bait. These intervals were used for calling variants, as sequence in these intervals without baits due to overlapping standard repeat masked regions could achieve coverage due to adjacent baits. There were 2,500,709 bases (2.5 Mb) covered by baits in 6,966 distinct intervals, and these bases were used when calculating coverage metrics.

### Pooled sequencing data analysis of 92 individuals at 464 genes

For pooled analysis, all reads from the indexed data set using captures of 22–24 individuals (Additional file 1: Table S1, rows 7–10) were considered, and the 7 bp indexes were trimmed from the end of read 1. All analysis was done using the SPLINTER6t package [33]. Forward and reverse reads of each pair were trimmed to 80 bp and analyzed as single reads. Each set of reads (forward and reverse) was compressed with “RAPGAP_read_compressor_v2.pl”. A reference sequence was created in fasta format consisting of the bait-covered intervals with 101 bp flanking regions joined with the PhiX174 reference sequence [34]. The compressed files were aligned with “RAPGAPHASH5d” to this reference sequence allowing up to five mismatches. Each aligned file was tagged with a file-specific identifier using “RAPGAP_alignment_tagger.pl” and the aligned files were concatenated together. An error model was then generated with “EMGENERATOR4” using the entire PhiX174 reference sequence, considering only unique reads, allowing up to five mismatches, and adding pseudocounts. The number of sequencing cycles to be incorporated was chosen as described by Vallania [9], with newer versions of SPLINTER allowing selective discarding of sequencing cycles with high cumulative error rates. The error model of the PhiX174 reads (Additional file 1: Figure S1) shows that cycles 3, 16, 20, 26, 39, 45, 47, 48, 49, 56, 57, 60, and 65 had cumulative error rates >0.02% in one of the two aligned files and were thus eliminated from variant calling. For variant calling with SPLINTER6t, the remaining 67 cycles were considered, up to five mismatches were allowed, the second order error model created with PhiX174 was utilized, a p-value cutoff of −1.3 (log10 scale) was used, and the allele count was set at 200. Coverage mode output was specified to allow coverage of all bases to be considered regardless of the presence of a variant.

Of the 92 individuals included in this data, 89 were successfully genotyped by the Illumina Human Omni 2.5-8 Beadchip SNV array. We restricted our comparison between the sequencing and the array by discarding array-based SNV positions as follows: 1) all alleles from the 89 successfully genotyped individuals (n = 178) did not contain a clear call, 2) if the position did not achieve an average of 20-fold coverage per allele in the sequencing data, 3) if the Illumina genotyping output was ambiguous as to the reference and variant allele, 4) if the position was a duplicate within the chip, in which case only one of the duplicate positions was considered. Of the 4,466 positions that overlapped between the array and our sequencing targets, 875 pass these filtering criteria. Of these, 376 were called wild type/wild type by the array in all individuals with no minor allele call by SPLINTER. The remaining 499 SNV positions (45,908 total) had at least one minor allele called by either the array or SPLINTER and were used to determine sequencing SNV concordance against the array.

For the 1 bp INDEL analysis, we compared the SPLINTER calls to an aggregate of the individual INDEL calls from the indexed analysis. We again restricted our comparisons to sites with at least 20-fold average coverage per allele, and additionally we considered only sites that were called as INDELs in the individual analysis.

### Pooled indexed custom capture data analysis

Sequencing analysis for indexed captures: Reads are separated by index, allowing for 1 bp mismatches. Reads are then aligned to the human genome (hg19/NCBI 37.0) with Novoalign (V2.07.15) using the parameters “-r none -e 1 -o softclip -H -k -t 200 -i PE 280 100 -a AGATCGGAAGAGCACAC GTCTGAACTCCAGTCACAGATCGGAAGAGCGTCGTGTAGGGAAAGATGTA” and output in SAM format with appropriate read group, ID, sample, platform, and library tags to conform to SAM specifications. Aligned files in SAM format are converted to compressed BAM format with SAMtools “view -bSu” and piped into samtools “sort”. All SAMtools commands were done with samtools-0.1.18. Duplicate reads are removed with picard-tools-1.58 “MarkDuplicates” using default settings and the resulting bam file is indexed by SAMtools. Reads are realigned around candidate INDELs with the Genome Analysis Toolkit’s (version 1.3-21) “RealignerTargetCreator” and “INDELRealigner” using default settings other than restricting the realignment to sequence within 1000 bp of our targeted intervals. Read pair information is fixed with picard-tools-1.58 “FixMateInformation”. This aligned file is indexed with SAMtools to yield the processed aligned file.

Variants are called using samtools-0.1.18 mpileup. We specify “-q 5 -Q15 -ABug”, as well as our targeted intervals with 50 bp flanking regions, and pipe the output into bcftools “view -bvcg”. This intermediate file is run through bcftools “view -e” and vcfutils “varFilter -D 1000”. We then change any heterozygous calls with under 20% of the reads matching the reference or variant allele to a homozygous variant genotype or homozygous wild type genotype, respectively.

We use the Novoalign metrics output to determine the number of reads that align and we use the Picard “MarkDuplicates” output to determine the percent duplication in our samples. We use the Genome Analysis Tookit’s version 1.0.2885 “DepthOfCoverage” to determine average coverage over specified intervals and utilize the histogram output to calculate the percent of bases reaching various coverage thresholds. We use picard-tools-1.58 “CalculateHSMetrics” to determine the percent of bases directly on our targeted intervals (on target), the percent of bases on and within 250 bp of our targeted intervals (on or near target), the fold enrichment for our captures, and the average coverage achieved by our captures. We specify for our target interval file and our base interval file a list of targets that were directly covered by baits.

Comparison of sequencing calls to array calls: The filtering criteria for choosing positions was identical to the pooled analysis, with two exceptions: 1) if an INDEL was called within 15 bp of an array position in at least one individual, that position was discarded, 2) no default coverage threshold was considered. In addition, if a SNV was called within 15 bp of an array position, that position was not considered for that individual. For the multiplexes of 22–24 individuals, 89 of 92 individuals had successful genotyping. For the multiplexes of 48 individuals, 94 of 96 individuals had successful genotyping. For the multiplexes of 30–32 individuals, all 92 individuals had successful genotyping. For each data set, we consider positions with 9 variant alleles or fewer as seen by the array to be rare variants. To calculate sensitivity, we sum the number of positions called heterozygous by the array and heterozygous by sequencing with the number of positions called homozygous variant by the array and homozygous variant by sequencing. We then divide this sum by the total number of positions called heterozygous or homozygous variant by the array. Thus, our sensitivity calculations are predicated on calling genotypes accurately, not solely determining the presence of a variant (i.e. if we call a site heterozygous and the array indicates it is homozygous variant, this counts against our sensitivity). To calculate specificity, we divide the number of positions called homozygous wild type by the array and homozygous wild type by sequencing by the total number of positions called homozygous wild type by the array.

### Sequencing

Sequencing was performed in the Center for Genome Sciences and Systems Biology at Washington University using either the Illumina Genome Analyzer IIx and generating 76 bp paired-end reads or the HiSeq 2000 platform generating 101 bp paired-end reads (Additional file 1: Table S1). Raw sequence data for the exome sequencing is available in dbGaP using accession number phs000553.v1.p1. GWA and sequencing information from the 280 LLFS patients is available in dbGaP using accession number phs000397.v1.p1.

### Genome wide microarray

Individuals undergoing exome sequencing were genotyped by the Affymetrix Genome-wide Human SNV Array 6.0 performed by the Washington University Clinical and Molecular Cytogenetics Laboratory. These data are accessible at dbGaP accession number phs000553.v1.p1. Individuals included in the custom pooled and indexed hybridization capture experiments were genotyped by the Illumina HumanOmni 2.5-8 Beadchip SNV array by the John’s Hopkin’s Center for Inherited Disease Research (CIDR). These data are accessible at dbGaP accession number phs000397.v1.p1.

## Abbreviations

MAF: Minor allele frequency; SNV: Single nucleotide variant; INDEL: Insertion or deletion; SPLINTER: Short INDEL Prediction by Large deviation Inference and Non-linear True frequency Estimation by Recursion; SPRI: Solid phase reversible immobilization; LLFS: Long Life Family Study; GWA: Genome wide array.

## Competing interests

The authors declare that they have no competing interests.

## Authors’ contributions

EIR participated in development of bioinformatics pipelines, data analysis, and drafting of the manuscript. BTL participated in library preparation, development of bioinformatics pipelines, data analysis and drafting the manuscript. SC, KT, AH, and AL assisted with library preparation. AY and FLMV assisted with data analysis for the pooled experiments. MP provided resources as well as participating in the data analysis and manuscript preparation. TED conceived of the study, participated in exome library preparation, data analysis and preparation of the manuscript. All authors read and approved the final manuscript.

## Authors’ information

Enrique Ramos and Benjamin T. Levinson regarded as joint First Authors.

## Supplementary Material

Additional file 1 **Additional Table 1.** A summary of the individuals, multiplexing, DNA input / person, hybridization reagents, sequencing platform, and use of indexing for the experiments described in this report. LLFS = Long Life Family Study participants. **Additional Table 2.** Individual and pooled exome sequencing metrics. Each of the five individual exomes was sequenced on 2 separate lanes of the Illumina Genome Analyzer (IIx) platform. Raw reads were aligned by Novoalign against the human NCBI reference sequence hg18 and variants were called by SAMtools using a ≥5-fold coverage threshold. As shown here, the amount of raw data and percentage of aligned reads was highly uniform for each independent sample. The pooled sample was sequenced on the Illumina HiSeq 2000 platform and aligned using Novoalign with only the exome reference target sequences with 76 bp flanks for each target. Given the difference in reference sequence, more raw data was discarded which artificially reduces the expected fold-enrichment. However, as evidenced by the mean target coverage of 1,389-fold (278-fold/person), we had more than ample data for variant calling at each target sequence. **Additional Figure 1.** SPLINTER error models generated in custom pooled sequencing analysis. These graphs model the percentage of sequencing errors (Y-axis) for each sequencing cycle (X-axis) for every type of substitution in the forward read (panel A) and the reverse read (panel B). The black line is the sum of all individual error rates. Similar plots were generated from 392 non-variant bases in the *FABP1* locus for the pooled exome experiment. Because this error modeling is done with each experiment, SPLINTER’s accuracy should remain consistent regardless of the Illumina sequencing platform used. Pr = probability (e.g. Pr (T∣C) means the probability of seeing an erroneous T given that the wild type base at that position is a C). **Additional Figure 2.** Using the NCBI CCDS exome definitions (http://www.ncbi.nlm.nih.gov/CCDS/CcdsBrowse.cgi), there are a maximum of 7,087 positions in common between the Agilent 38 Mb exome and Affymetrix 6.0 GWA. Due to “no-calls” on either platform, there was an average of 6,725 positions/individual used to calculate sensitivity and specificity. Sequencing calls were made using a coverage threshold of 5-fold/chromosome. For pooled exome sequencing, we generated 1.44x10^9^ total paired-end reads, of which 66.82% aligned to the annotated reference. At 76 bp per read, this equates to an average of 192-fold coverage of every base in the 38 Mb capture per allele in our 10 allele pool. Aligned reads in “Native” format from Novoalign were converted to a format compatible with SPLINTER for SNV calling using a custom Perl script (found online at http://druleylab.wustl.edu). A) Sensitivity for individual and pooled exome variant calling as compared to an average of 2,937 true variant positions detected by Affymetrix 6.0 array. To be considered accurate, copy number must match (i.e. heterozygous vs. homozygous) between the array and the sequencing. The average sensitivity (defined as identifying the same genotype) is 97% for single exomes and 99.4% for the pooled sample. The pooled sequencing compares an aggregate of minor allele frequencies from the array data against the SPLINTER calls for these variants. SPLINTER called 2,937 variant positions (of 6,725). B) Specificity was determined by comparing the remaining wild-type array positions, an average of 4,337 for each sample, to sequencing calls. The average specificity across all five individual exomes was 99.8%. Specificity for the pooled sample (99.7%) is calculated by dividing the number of true negative base calls (by array and sequencing; N = 3,788) by the sum of the number of true negatives and false positives (wild type by array, but variant by sequencing). **Additional Table 3.** Individually genotyped variant positions from pooled exome sequencing. The asterisk (*) denotes that in this pool of 4 females and 1 male, we assumed nine X-chromosomes and the minor allele frequencies are rounded accordingly. There were a total of 127 variant positions not-included on the genome-wide array that were individually validated by individual genotyping using Sequenom MassArray. Six of these positions were novel variants not found in dbSNP 132. NC = no call made by the SPLINTER algorithm. **Additional Figure 3.** Discordance between single and pooled exome minor allele frequency estimates is correlated to a lack of sequencing coverage. The 2,937 variant positions analyzed in Figure 1 were graphed against the relative coverage per variant position (vertical axis). The broader base of the plot (dark blue cubes) demonstrates that outliers are most likely due to a lack of sequencing coverage in either the individual or pooled sequencing rather than the introduction of systemic false positive artifacts. **Additional Table 4.** Candidate genes chosen for resequencing from the Long Life Family Study (LLFS). These 464 genes were chosen by LLFS investigators for resequencing based on published data relevant to the study aims. **Additional Figure 4.** Concordance in INDEL identification between pooled custom capture sequencing analysis by SPLINTER and SAMtools from the same indexed individuals. Of the 110 INDELS called with ≥20-fold coverage in individual analysis, we identified 99 (90%) with SPLINTER. For rare INDEL calling, 54 of the 110 were found at a frequency of <5%, and SPLINTER detected 45 (83.3%). **Additional Table 5.** Blockers for hybridization. **Additional Figure 5.** Raw and aligned read uniformity. The raw and aligned, unique read counts per individual in four hybridizations of 22–24 individuals (Additional Table 1, rows 7–10) and two hybridizations of 48 individuals (Additional Table 1, rows 14–15). Individuals are ranked from lowest total read count to highest total read count by total reads per person (black and dark blue symbols). Dark and light blue symbols: the open squares, circles, triangles, and closed circles indicate the four different captures of 22–24 individuals. Black and gray symbols: the open squares and circles indicate the two captures of 48 individuals each. The distribution of symbols across each dataset demonstrates that individual results did not cluster within captures. The table lists the metrics for each lane of sequencing, the fold difference between individuals within each lane and the percentage of indexes identifiable within each raw data set. **Additional Figure 6.** PCR duplicates and capture efficiency for multiplexes of 22–24 (Additional Table 1, rows 7–10). The graph shows the individual percent of: PCR duplicates (red symbols), aligned data on target (green symbols) and aligned data on and near targeted intervals (blue symbols). The two samples with very low read counts are not shown and the rest of the samples (n=90) are ranked from lowest total read count to highest total read count. Different shapes and filling indicate the four independent captures that were combined for sequencing. “On and near target” is defined as bases that align on or within 250 bp of target intervals after removing duplicates. “On target” is defined as bases that align directly on targeted intervals after removing duplicates. The “Individual samples” numbers correspond to the ones used in Additional Figure 3. **Additional Figure 7.** Targeted intervals ranked by coverage achieved between different samples and captures. One sample from each of the four multiplexes of 22–24 was chosen for this plot. Observed indexes ACAGATA, ATCAGCA, TGCTGGG, TTACGAT where chosen, with read counts ranging from (1,580,586 - 1,599,797). Of the 6,966 distinct targeted intervals, the 6,604 that achieved greater than 0x average coverage in all 4 of these samples were considered for this plot. All intervals were ranked by average coverage within each sample with 1 being the highest and 6,604 being the lowest and plotted. Between all four samples, the average pairwise *R*^2^ = 0.936, showing a high level of uniformity between captures. To determine if candidate regions were enriched uniformly across the four multiplexes, we ranked the 6,966 intervals that were covered by baits by coverage in one sample from each of the four captures. Comparing the ranks of all 6,604 intervals with over >0X coverage in each of the four samples, the average pairwise *R*^2^ = 0.936 (Additional Figure 4). Thus, our method performs uniformly with respect to both aggregate metrics of an entire capture as well as bait-to-bait capture efficiency. **Additional Figure 8.** PCR duplicates and capture efficiency for multiplexes of 48 (Additional Table 1, rows 14–15). The graph shows the individual percent of: PCR duplicates (red symbols), aligned data on target (green symbols) and aligned data on and near targeted intervals (blue symbols). The two samples with very low read counts are not shown and the rest of the samples (n=90) are ranked from lowest total read count to highest total read count. Different shapes indicate the two independent captures that were combined for sequencing. “On and near target” is defined as bases that align on or within 250 bp of target intervals after removing duplicates. “On target” is defined as bases that align directly on targeted intervals after removing duplicates. The “Individual samples” numbers correspond to the ones used in Additional Figure 3. We see an improvement in overall on/near target percentage compared to our smaller multiplexing experiments due to improving our blocking strategy by adding 1 uL of the 58 bp post-hybridization PCR primer Illumina PE 1.0 (the longer oligo listed under Post-hybridization PCR primers on Additional Table 5) to the Hybridization Capture step outlined in the Supplemental Methods. **Additional Figure 9.** Comparing genome-wide array position coverage to sequencing coverage. This plot was generated using the observed index TAGTATT from the set of 22–24 multiplexed captures, which achieved 1,404,000 million reads, closest to the average for all 92 samples. Blue circles: percent of targeted bases at specified coverage threshold. Red circles: percent of array positions at specified coverage thresholds. Squares: median coverage achieved when considering only positions at 3-fold coverage or greater. Triangles: mean coverage achieved when considering only positions at 3-fold coverage or greater. The close overlap of the mean and median indicate the array-based positions accurately reflect the sequencing positions with respect to the overall coverage achieved. **Additional Figure 10A.** Coverage achieved depends on GC content of designed baits. This plot was generated using the observed index TAGTATT from the set of 22–24 multiplexed captures, which achieved 1,404,000 million reads, closest to the average for all 92 samples. The average coverage for each bait was plotted against the% GC content of the bait with the boxplot function in R. **Additional Figure 10B.** Coverage achieved depends on target interval size. This plot was also generated using the observed index TAGTATT. The average coverage for each bait was plotted against the target interval size that the bait belonged to with the boxplot function in R. Higher coverage in targeted intervals ≥240 bp was observed relative to 120 bp and 180 bp intervals. While partially due to having a greater number of bases targeted by two baits in these intervals due to specifying 2X tiling frequency, we feel this is more largely a result of flanking sequence from a fragment for one bait yielding coverage for an adjacent bait. **Additional Figure 11.** Bioanalyzer traces of subsequent steps during indexed library preparation. Red line: individual sonicated and purified sample. Blue line: individual end-repaired and purified sample. Green line: individual adapter ligated and purified sample. The three different populations beyond 100bp may indicate fragments with 0, 1, and 2 adapters ligated. The bimodal peaks <100 bp correspond to unligated adapters. Cyan line: pre-capture, purified PCR product. Magenta line: post-capture, purified PCR product. The fact that the majority of our fragment sizes were >200 bp supports our previous conclusion that our fragments of ≥240 bp demonstrated higher total coverage due to flanking sequence from one bait contributing to total coverage for an adjacent bait (see Additional Figure 10B). **Additional Figure 12.** Strand bias is not adversely affecting sequencing calls. To explore whether strand bias in sequencing output was adversely affecting variant calls in the custom capture sequencing from the multiplexes of 48 individuals (Additional Table 1, rows 14–15), we summed all variant calls from all 96 individuals and compared the total number of positions (Y-axis) against the percentage of raw sequencing reads generated from the forward strand (X-axis). Each column represents a “bin” of a given number of variants with a given percentage of raw reads generated from the forward strand. We performed this comparison for all called variants, known and novel, at all MAFs (Panel A; n = 148,972), against the gold standard dataset of all positions called homozygous wild-type by GWA (Panel B; n = 308,842). The two profiles are very similar and the central peak indicates that the majority of positions had similar amounts of raw data from each strand, suggesting that strand bias is not adversely affecting sequencing calls for all positions not called homozygous wild-type by GWA. **Additional Table 6.** The percentage of misattributed indexes is improved by an additional purification of unligated adapter sequences. To test whether unligated adapters were the source of misattributed indexes in the multiplexes of 48 (Additional Table 1, rows 14–15), we implemented an additional purification with 9.45% PEG and 1.25M NaCl after pooling the adapter-ligated samples and prior to the subsequent PCR enrichment to more thoroughly remove any unligated adapters in the multiplexes of 30–32 (Additional Table 1, rows 11–13). We focused on positions with a MAF around 50% (range 39-59%), which provides more positions to query (215,580 reads mapped to wild type alleles, 3,828 reads mapped to variant alleles) where multiple individuals should possess one of three distinct genotypes (AA, AB, BB). Of the 96 individuals captured in two multiplexes of 48, 94 had valid array genotyping. All positions with 188 valid allele calls were binned according to the number of GWA-called variant alleles in the cohort. Of the 92 individuals captured in three multiplexes of 30–32, all 92 had valid array genotyping. Analysis was identical to the multiplexes of 48, with two samples excluded due to low coverage and one sample excluded due to having a very high mismatch rate, possibly due to genotyping performing poorly or a sample handling mishap not indicative of the method as a whole. Introducing the extra purification step improved the percentage of misattributed indexes. With the additional purification in place, we find an average of 1.8% of variant reads that are attributed to an individual who was homozygous wild type by array. At base positions where no variant alleles were called by array, 0.13% of reads erroneously contain a sequence variant, likely due to sequencing error or alignment artifacts. Subtracting this background from the 1.8% of misattributed indexes at positions with a validated variant in approximately half of all alleles yields 1.67%. We assume that this index switching is a random and stochastic process, suggesting that we only “see” half of reads with an inappropriate index because the other half would adjoin with a read having a matching genotype (e.g. instead of an index switching from a wild type read to a variant read, it switches from a wild type read to another wild type read). Thus, we conclude that with the additional purification a total of ~3.4% of reads contain misattributed indexes. **Additional Figure 13.** Schematic diagram demonstrating how indexed adapters may become affixed to a different source molecule. A) Primers, Y-shaped adapter-ligated fragments, and unindexed ligated adapters are mixed together prior to PCR amplification. B) After denaturation during the first PCR cycle, the reverse primer sits down on its complementary site during the annealing step. The non-indexed strand of the unligated Y-shaped adapter is not shown. C) During the extension phase of the first PCR cycle, the reverse complement strand of the template is synthesized. D) After denaturation during the second PCR cycle, the primers sit down on their complementary site during the annealing step. Additionally, any unligated adapter can sit down and serve as a primer. An indexing strand with a different index is shown. E) During the extension phase of the second PCR cycle, any individual-specific sequencing alterations can be attributed to incorrect indexes. F) After the second PCR cycle, the strand attributed to the wrong index can be amplified exponentially. **Supplemental Methods.** We are starting with 70-375ng of purified genomic DNA per person in the protocol, suspended in 15ul of TE. [Note: For multiplexes of 48, we have started with 350 ng, 280 ng, 210 ng, 140 ng, and 70 ng of DNA/person and achieved equivalent results in terms of percent duplication, data on target, coverage achieved, sensitivity, and specificity (data not shown).] Click here for file

## References

[B1] Rivas MA Beaudoin M Gardet A Stevens C Sharma Y Zhang CK Boucher G Ripke S Ellinghaus D Burtt N Fennell T Kirby A Latiano A Goyette P Green T Halfvarson J Haritunians T Korn JM Kuruvilla F Lagace C Neale B Lo KS Schumm P Torkvist L Dubinsky MC Brant SR Silverberg MS National Institute of Diabetes and Digestive Kidney Diseases Inflammatory Bowel Disease Genetics Consortium (NIDDK IBDGC) United Kingdom Inflammatory Bowel Disease Genetics Consortium, International Inflammatory Bowel Disease Genetics Consortium Deep resequencing of GWAS loci identifies independent rare variants associated with inflammatory bowel disease Nat Genet 2011 43 1066 1073 10.1038/ng.952 21983784 PMC3378381

[B2] Cohen JC Kiss RS Pertsemlidis A Marcel YL McPherson R Hobbs HH Multiple rare alleles contribute to low plasma levels of HDL cholesterol Science 2004 305 869 872 10.1126/science.1099870 15297675

[B3] Ahituv N Kavaslar N Schackwitz W Ustaszewska A Martin J Hebert S Doelle H Ersoy B Kryukov G Schmidt S Medical sequencing at the extremes of body mass Am J Hum Genet 2007 80 779 791 10.1086/513471 17357083 PMC1852707

[B4] Li Y Vinckenbosch N Tian G Huerta-Sanchez E Jiang T Jiang H Albrechtsen A Andersen G Cao H Korneliussen T Resequencing of 200 human exomes identifies an excess of low-frequency non-synonymous coding variants Nat Genet 2010 42 969 972 10.1038/ng.680 20890277

[B5] Ng SB Buckingham KJ Lee C Bigham AW Tabor HK Dent KM Huff CD Shannon PT Jabs EW Nickerson DA Shendure J Bamshad MJ Exome sequencing identifies the cause of a Mendelian disorder Nat Genet 2010 42 30 35 10.1038/ng.499 19915526 PMC2847889

[B6] Bilvugar K Ozturk AK Louvi A Kwan KY Choi M Tatli B Yalnizoglu D Tuysuz B Caglayan AO Gokben S Whole-exome sequencing identifies recessive WDR62 mutations in severe brain malformations Nature 2010 467 207 210 10.1038/nature09327 20729831 PMC3129007

[B7] Ng SB Bigham AW Buckingham KJ Hannibal MC McMillin MJ Gildersleeve HI Beck AE Tabor HK Cooper GM Mefford HC Exome sequencing identifies *MLL2* mutations as a cause of Kabuki syndrome Nat Genet 2010 42 790 793 10.1038/ng.646 20711175 PMC2930028

[B8] Druley TE Vallania FL Wegner DJ Varley KE Knowles OL Bonds JA Robison SW Doniger SW Hamvas A Cole FS Fay JC Mitra RD Quantification of rare allelic variants from pooled genomic DNA Nat Methods 2009 6 263 265 10.1038/nmeth.1307 19252504 PMC2776647

[B9] Vallania FL Druley TE Ramos E Wang J Borecki I Province M Mitra RD High-throughput discovery of rare insertions and deletions in large cohorts Genome Res 2010 20 1711 1718 available at http://www.ibridgenetwork.org/wustl/splinter. 10.1101/gr.109157.110 21041413 PMC2989997

[B10] Day-Williams AG McLay K Drury E Edkins S Coffey AJ Palotie A Zeggini E An evaluation of different target enrichment methods in pooled sequencing designs for complex disease association studies PLoS One 2011 6 e26279 10.1371/journal.pone.0026279 22069447 PMC3206031

[B11] Harakalova M Nijman IJ Medic J Mokry M Renkens I Blankensteijn JD Kloosterman W Baas AF Cuppen E Genomic DNA pooling strategy for next-generation sequencing-based rare variant discovery in abdominal aortic aneurysm regions of interest – challenges and limitations J Cardiovasc Trans Res 2011 4 271 280 10.1007/s12265-011-9263-5 PMC3099005 21360310

[B12] Cummings N King R Rickers A Kaspi A Lunke S Haviv I Jowett JBM Combining target enrichment with barcode multiplexing for high throughput SNP discovery BMC Genomics 2010 11 641 10.1186/1471-2164-11-641 21083938 PMC3012606

[B13] Kenny EM Cormican P Gilks WP Gates AS O’Dushlaine CT Pinto C Corvin AP Gill M Morris DW Multiplex target enrichment using DNA indexing for ultra-high throughput SNP detection DNA Res 2011 18 31 38 10.1093/dnares/dsq029 21163834 PMC3041504

[B14] Wesolowska A Dalgaard MD Borst L Gautier L Bak M Weinhold N Nielsen BF Helt LR Audouze K Nersting J Cost-effective multiplexing before capture allows screening of 25,000 clinically relevant SNPs in childhood acute lymphoblastic leukemia Leukemia 2011 25 1001 1006 10.1038/leu.2011.32 21415851

[B15] Nijman IJ Mokry M van Boxtel R Toonen P de Bruijn E Cuppen E Mutation discovery by targeted genomic enrichment of multiplexed barcoded samples Nat Methods 2010 7 913 915 10.1038/nmeth.1516 20953175

[B16] Rohland N Reich D Cost-effective, high-throughput DNA sequencing libraries for multiplexed target capture Gen Research 2012 22 939 946 10.1101/gr.128124.111 PMC3337438 22267522

[B17] Harakalova M Mokry M Hrdlickova B Renkens I Duran K van Roekel H Lansu N van Roosmalen M de Bruijn E Nijman IJ Multiplexed array-based and in-solution genomic enrichment for flexible and cost-effective targeted next-generation sequencing Nat Protocols 2011 6 1870 1886 10.1038/nprot.2011.396 22051800

[B18] Bansal V Tewhey R Leproust EM Schork NJ Efficient and cost effective population resequencing by pooling and in-solution hybridization PLoS One 2011 6 e18353 10.1371/journal.pone.0018353 21479135 PMC3068187

[B19] Fisher S Barry A Abreu J Minie B Nolan J Delorey TM Young G Fennell TJ Allen A Ambrogio L Berlin AM Blumenstiel B Cibulskis K Friedrich D Johnson R Juhn F Reilly B Shammas R Stalker J Sykes SM Thompson J Walsh J Zimmer A Zwirko Z Gabriel S Nicol R Nusbaum C A scalable, fully automated process for construction of sequence-ready human exome targeted capture libraries Genome Biol 2011 12 R1 10.1186/gb-2011-12-1-r1 21205303 PMC3091298

[B20] Agilent SureSelect Target Enrichmenthttp://www.genomics.agilent.com.

[B21] MAQ: Mapping and Assembly with Qualityhttp://maq.sourceforge.net.

[B22] Cross_matchhttp://www.phrap.org.

[B23] Li H Handsaker B Wysoker A Fennell T Ruan J Homer N Marth G Abecasis G Durbin R The Sequence Alignment/Map format and SAMtools Bioinformatics 2009 25 2078 2079 http://samtools.sourceforge.net. 10.1093/bioinformatics/btp352 19505943 PMC2723002

[B24] Sequenom MassArray http://hg.wustl.edu/info/Sequenom_description.html.

[B25] NHGRI’s Exome Variant Server, Support Vector Machine http://evs.gs.washington.edu/EVS/; using February 2012 data.

[B26] DePristo MA Banks E Poplin R Garimella KV Maguire JR Hartl C Philippakis AA del Angel G Rivas MA Hanna M McKenna A Fennell TJ Kernytsky AM Sivachenko AY Cibulskis K Gabriel SB Altshuler D Daly MJ A framework for variation discovery and genotyping using next-generation DNA sequencing data Nat Genet 2011 43 491 498 10.1038/ng.806 21478889 PMC3083463

[B27] Kircher M Sawyer S Meyer M Double indexing overcomes inaccuracies in multiplex sequencing on the Illumina platform Nucleic Acids Res 2012 40 e3 10.1093/nar/gkr771 22021376 PMC3245947

[B28] NCBI Human Genome Reference Sequence, Build 36 (hg18) http://www.ncbi.nlm.nih.gov/mapview/stats/BuildStats.cgi?taxid=9606&build=36&ver=3.

[B29] Novoalign, license purchased from Novocraft Technologieshttp://www.novocraft.com.

[B30] Druley lab, script for converting Novoalign output to SPLINTER variant calling formathttp://druleylab.wustl.edu/ under the “Projects: Pooled hybridization capture with indexing” tab.

[B31] Long Life Family Studyhttp://www.longlifefamilystudy.org/.

[B32] Agilent eArray hybridization capture bait design toolhttps://earray.chem.agilent.com/earray/.

[B33] SPLINTER6t bioinformatics package updateavailable at http://www.genetics.wustl.edu/rmlab.

[B34] PhiX174 reference sequencehttp://www.ncbi.nlm.nih.gov/nuccore/9626372?report=fasta.

