# Supplementary material for: Population-based rare variant detection via pooled exome or custom hybridization capture with or without individual indexing
Source: BMC Genomics. 2012 Dec 6;13:683. doi: 10.1186/1471-2164-13-683 (PMC3534616; doi:10.1186/1471-2164-13-683)
Supplement: Additional file 1 — Additional Table 1. A summary of the individuals, multiplexing, DNA input / person, hybridization reagents, sequencing platform, and use of indexing for the experiments described in this report. LLFS = Long Life Family Study participants. Additional Table 2. Individual and pooled exome sequencing metrics. Each of the five individual exomes was sequenced on 2 separate lanes of the Illumina Genome Analyzer (IIx) platform. Raw reads were aligned by Novoalign against the human NCBI reference sequence hg18 and variants were called by SAMtools using a ≥5-fold coverage threshold. As shown here, the amount of raw data and percentage of aligned reads was highly uniform for each independent sample. The pooled sample was sequenced on the Illumina HiSeq 2000 platform and aligned using Novoalign with only the exome reference target sequences with 76 bp flanks for each target. Given the difference in reference sequence, more raw data was discarded which artificially reduces the expected fold-enrichment. However, as evidenced by the mean target coverage of 1,389-fold (278-fold/person), we had more than ample data for variant calling at each target sequence. Additional Figure 1. SPLINTER error models generated in custom pooled sequencing analysis. These graphs model the percentage of sequencing errors (Y-axis) for each sequencing cycle (X-axis) for every type of substitution in the forward read (panel A) and the reverse read (panel B). The black line is the sum of all individual error rates. Similar plots were generated from 392 non-variant bases in the FABP1 locus for the pooled exome experiment. Because this error modeling is done with each experiment, SPLINTER’s accuracy should remain consistent regardless of the Illumina sequencing platform used. Pr = probability (e.g. Pr (T∣C) means the probability of seeing an erroneous T given that the wild type base at that position is a C). Additional Figure 2. Using the NCBI CCDS exome definitions (http://www.ncbi.nlm.nih.gov/CCDS/CcdsBro [file 1471-2164-13-683-S1.pdf]

Additional Data and Methods

Population-based rare variant detection via pooled exome or custom hybridization capture with or without individual indexing.

Enrique Ramos*[1], Benjamin T. Levinson*[1], Sara Chasnoff[1], Andrew Hughes[1], Andrew Young[1], Katherine Thornton[1], Allie Li[2], Francesco M.L. Vallania[1], Michael Province[1], Todd E. Druley[†1,3].

<table>
<tr><th></th><th>Sample</th><th>Number of Individuals</th><th>Input DNA per person</th><th>Hybridization Kit</th><th>Sequencing Platform</th><th>Batched for Sequencing</th><th colspan="2">Indexing</th></tr>
<tr><td>1</td><td>Individual Exome 1</td><td>1</td><td>3 µg</td><td rowspan="5">Agilent SureSelect Human All Exon 38Mb</td><td rowspan="5">Illumina GAIIx</td><td>No</td><td colspan="2">No</td></tr>
<tr><td>2</td><td>Individual Exome 2</td><td>1</td><td>3 µg</td><td>No</td><td colspan="2">No</td></tr>
<tr><td>3</td><td>Individual Exome 3</td><td>1</td><td>3 µg</td><td>No</td><td colspan="2">No</td></tr>
<tr><td>4</td><td>Individual Exome 4</td><td>1</td><td>3 µg</td><td>No</td><td colspan="2">No</td></tr>
<tr><td>5</td><td>Individual Exome 5</td><td>1</td><td>3 µg</td><td>No</td><td colspan="2">No</td></tr>
<tr><td>6</td><td>Pooled Exomes</td><td>5</td><td>500 ng</td><td>Agilent SureSelect Human All Exon 38Mb</td><td>Illumina HiSeq 2000</td><td>N = 5</td><td colspan="2">No</td></tr>
<tr><td>7</td><td>LLFS</td><td>22</td><td>350 ng</td><td rowspan="4">Agilent SureSelect Custom Hybridization Capture</td><td rowspan="4">Illumina HiSeq 2000</td><td rowspan="4">N = 92</td><td>Yes</td><td rowspan="4">No (for pooled expt)</td></tr>
<tr><td>8</td><td>LLFS</td><td>23</td><td>350 ng</td><td>Yes</td></tr>
<tr><td>9</td><td>LLFS</td><td>23</td><td>350 ng</td><td>Yes</td></tr>
<tr><td>10</td><td>LLFS</td><td>24</td><td>350 ng</td><td>Yes</td></tr>
<tr><td>11</td><td>LLFS</td><td>30</td><td>350 ng</td><td rowspan="3">Agilent SureSelect Custom Hybridization Capture</td><td rowspan="3">Illumina HiSeq 2000</td><td rowspan="3">N = 92</td><td colspan="2">Yes</td></tr>
<tr><td>12</td><td>LLFS</td><td>30</td><td>350 ng</td><td colspan="2">Yes</td></tr>
<tr><td>13</td><td>LLFS</td><td>32</td><td>350 ng</td><td colspan="2">Yes</td></tr>
<tr><td>14</td><td>LLFS</td><td>48</td><td>70-350 ng</td><td rowspan="2">Agilent SureSelect Custom Hybridization Capture</td><td rowspan="2">Illumina HiSeq 2000</td><td rowspan="2">N = 96</td><td colspan="2">Yes</td></tr>
<tr><td>15</td><td>LLFS</td><td>48</td><td>70-350 ng</td><td colspan="2">Yes</td></tr>
</table>

Additional Table 1. A summary of the individuals, multiplexing, DNA input / person, hybridization reagents, sequencing platform, and use of indexing for the experiments described in this report. LLFS = Long Life Family Study participants.

Todd Druley 11/7/12 9:25 AM
Formatted: No underline

	Total Raw Reads	Aligned reads	Aligner / SNP caller	Reference sequence	Sequencing platform	Percent Aligned	Mean Target Coverage	On-target % Bases	Fold-enrichment	Percent of exome covered	Total Variants	Novel Variants
Person 1	141,082,854	115,958,854	Novoalign / SAMtools	hg18 genome	Illumina GAIIx	82.20	90.07	46.32	31.10	99.40	16,186	1,284
Person 2	139,228,760	115,448,532	Novoalign / SAMtools	hg18 genome	Illumina GAIIx	82.90	100.05	52.70	35.43	99.58	16,086	1,132
Person 3	136,806,126	113,249,996	Novoalign / SAMtools	hg18 genome	Illumina GAIIx	82.80	92.47	50.26	33.36	99.47	16,095	1,073
Person 4	139,614,682	108,541,251	Novoalign / SAMtools	hg18 genome	Illumina GAIIx	77.70	89.14	48.07	32.35	99.34	16,009	1,277
Person 5	141,144,750	111,436,144	Novoalign / SAMtools	hg18 genome	Illumina GAIIx	79.00	86.15	47.81	32.04	99.28	16,040	1,204
TOTAL	697,877,172	564,634,777									80,416	5,970
AVERAGE	139,575,434	112,926,955				80.92	91.58	49.03	32.86	99.41	16,083	1,194
Pooled Sample	1,439,982,960	777,546,928	Novoalign / SPLINTER	Exome bait reference with 76bp flanking regions (hg18)	Illumina HiSeq 2000	53.99	1,389.05	89.08	1.48	99.99	23,110	7,633

Additional Table 2. Individual and pooled exome sequencing metrics. Each of the five individual exomes was sequenced on 2 separate lanes of the Illumina Genome Analyzer (IIx) platform. Raw reads were aligned by Novoalign against the human NCBI reference sequence hg18 and variants were called by SAMtools using a ≥5-fold coverage threshold. As shown here, the amount of raw data and percentage of aligned reads was highly uniform for each independent sample. The pooled sample was sequenced on the Illumina HiSeq 2000 platform and aligned using Novoalign with only the exome reference target sequences with 76 bp flanks for each target. Given the difference in reference sequence, more raw data was discarded which artificially reduces the expected fold-enrichment. However, as evidenced by the mean target coverage of 1,389-fold (278-fold/person), we had more than ample data for variant calling at each target sequence.

Todd Druley 11/7/12 9:25 AM
Formatted: No underline

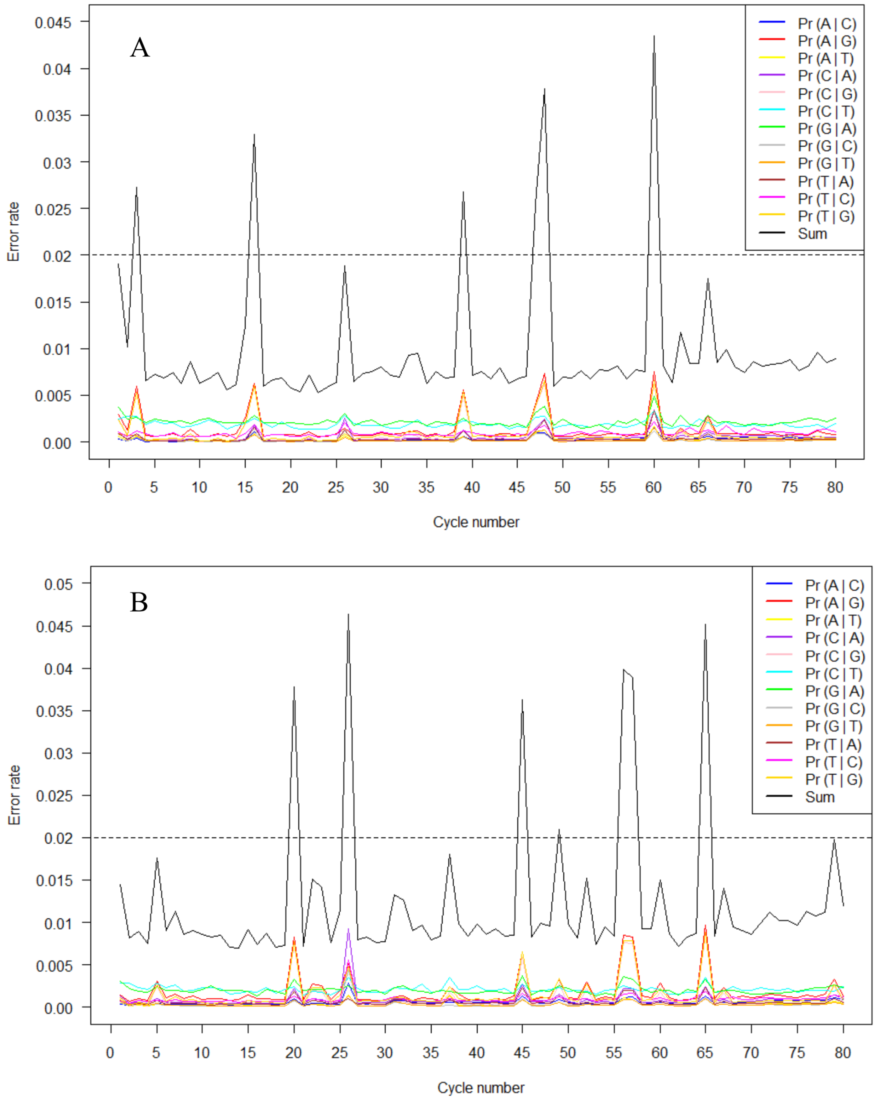

Additional Figure 1. SPLINTER error models generated in custom pooled sequencing analysis. These graphs model the percentage of sequencing errors (Y-axis) for each sequencing cycle (X-axis) for every type of substitution in the forward read (panel A) and the reverse read (panel B). The black line is the sum of all individual error rates. Similar plots were generated from 392 non-variant bases in the *FABP1* locus for the pooled exome experiment. Because this error modeling is done with each experiment, SPLINTER's accuracy should remain consistent regardless of the Illumina sequencing platform used. Pr = probability (e.g. Pr (T|C) means the probability of seeing an erroneous T given that the wild type base at that position is a C).

Todd Druley 11/7/12 9:25 AM
Formatted: No underline

Todd Druley 11/7/12 9:25 AM
Formatted: No underline

Todd Druley 11/7/12 9:25 AM
Formatted: No underline

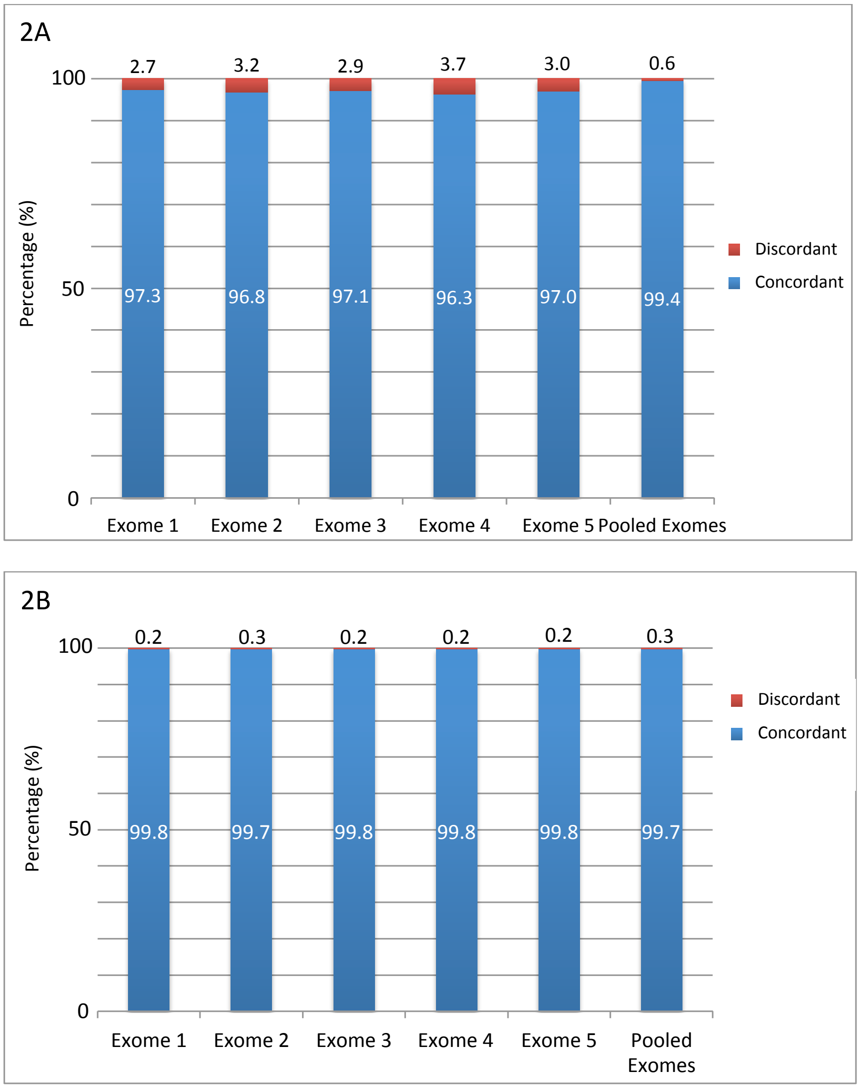

Additional Figure 2.

Using the NCBI CCDS exome definitions (http://www.ncbi.nlm.nih.gov/CCDS/CcdsBrowse.cgi), there are a maximum of 7,087 positions in common between the Agilent 38 Mb exome and Affymetrix 6.0 GWA. Due to "no-calls" on either platform, there was an average of 6,725 positions/individual used to calculate sensitivity and specificity. Sequencing calls were made using a coverage threshold of 5-fold/chromosome. For pooled exome sequencing, we generated $1.44x10^9$ total paired-end reads, of which 66.82% aligned to the annotated reference. At 76 bp per read, this equates to an average of 192-fold coverage of every base in the 38 Mb capture per allele in our 10 allele pool. Aligned reads in "Native"

Todd Druley 11/7/12 9:25 AM
Formatted: No underline

Todd Druley 11/7/12 9:25 AM
Formatted: Default Paragraph Font, Font:Calibri

Todd Druley 11/7/12 9:25 AM
Formatted: No underline

format from Novoalign were converted to a format compatible with SPLINTER for SNV calling using a custom Perl script (found online at http://druleylab.wustl.edu).

A) Sensitivity for individual and pooled exome variant calling as compared to an average of 2,937 true variant positions detected by Affymetrix 6.0 array. To be considered accurate, copy number must match (i.e. heterozygous vs. homozygous) between the array and the sequencing. The average sensitivity (defined as identifying the same genotype) is 97% for single exomes and 99.4% for the pooled sample. The pooled sequencing compares an aggregate of minor allele frequencies from the array data against the SPLINTER calls for these variants. SPLINTER called 2,937 variant positions (of 6,725)

B) Specificity was determined by comparing the remaining wild-type array positions, an average of 4,337 for each sample, to sequencing calls. The average specificity across all five individual exomes was 99.8%. Specificity for the pooled sample (99.7%) is calculated by dividing the number of true negative base calls (by array and sequencing; N = 3,788) by the sum of the number of true negatives and false positives (wild type by array, but variant by sequencing).

Todd Druley 11/7/12 9:25 AM
Formatted: Default Paragraph Font, Font:Calibri

Todd Druley 11/7/12 9:25 AM
Formatted: No underline

Chr	Position (hg18)	dbSNP (build 132)	Major Allele	Minor Allele	Sequenom MAF	SAMtools MAF	SPLINTER MAF
1	65856282	novel	G	A	0.4	0.4	0.3
1	86682170	rs1413426	G	A	0.7	0.7	0.7
1	178153748	rs627897	G	A	1	1	1
1	84885791	rs2994949	T	C	1	1	1
1	94416119	rs1048854	T	C	0.4	0.4	0.4
1	97937679	rs2297595	T	C	0.8	0.8	0.8
1	157253101	rs866484	G	C	0.5	0.5	0.5
1	208036525	rs7552506	G	C	0.3	0.3	0.2
1	1148494	rs6603781	A	G	0.6	0.6	0.5
1	15706947	rs2020902	A	G	0.3	0.3	0.3
1	110033269	novel	A	G	0.8	0.8	1
1	183402368	rs950327	A	G	0.4	0.4	0.4
1	150648744	rs3829868	C	T	0.4	0.4	0.4
1	156717006	rs1418843	C	T	0.9	0.9	0.9
1	159284180	rs2774279	C	T	0.5	0.5	0.4
1	203319842	rs4951168	C	T	1	1	1
2	190028328	rs6737615	C	A	0.4	0.4	0.4
2	43958429	rs6544718	T	C	0.5	0.5	0.5
2	196381778	rs13411834	A	G	1	1	1
2	27293255	rs7437	G	T	0.3	0.3	0.3
2	48662402	rs3749145	C	T	0.5	0.5	0.5
2	96162032	rs56238667	C	T	0.2	0.2	0.1
2	113387849	rs3811046	G	T	0.5	0.5	0.5
2	158335226	rs1146031	C	T	1	1	1
2	169741235	rs2229265	C	T	0.5	0.5	0.4
3	188571620	rs1533594	G	A	0.8	0.8	0.8
3	191194704	rs1719600	G	A	0.8	0.8	0.8
3	4999771	rs908078	T	C	0.2	0.2	0.1
3	110535422	rs3762648	T	C	0.5	0.5	0.5
3	113467797	rs9872691	T	C	0.3	0.3	0.3
3	188400445	rs6764714	C	G	0.5	0.5	0.6
4	122961667	rs769242	T	C	1	1	1
4	2145531	rs2022302	A	G	0.4	0.4	0.4
4	96475639	rs4699423	A	G	0.7	0.7	0.7
4	110838983	rs2301717	T	G	0.7	0.7	NC
4	114497726	rs3733617	C	T	0.3	0.3	0.3

Chr	Position (hg18)	dbSNP (build 132)	Major Allele	Minor Allele	Sequenom MAF	SAMtools MAF	SPLINTER MAF
5	133915679	rs12163993	G	A	0.5	0.5	0.4
5	150866918	rs7723266	G	A	1	1	1
5	170171690	rs1063310	C	A	0.5	0.5	0.5
5	3653334	rs844154	T	C	0.8	0.8	0.7
5	52193131	rs2447867	T	C	0.7	0.7	0.7
5	101844068	rs17151033	C	T	0.2	0.2	NC
6	152307215	rs1801132	G	C	0.5	0.5	0.5
6	160927528	rs7765803	G	C	1	1	1
6	56592717	rs2230862	C	T	0.9	0.9	0.9
6	152725106	rs4407724	G	T	1	1	1
7	123056354	rs4731112	G	C	1	1	1
7	133865943	rs1722883	T	C	1	1	1
7	134500117	rs2288237	A	C	0.8	0.7	0.7
7	73571810	novel	C	T	0.3	0.3	0.2
7	92602425	rs10488532	C	T	0.3	0.3	0.3
8	6780991	rs2738100	A	G	0.4	0.4	0.3
8	144803561	rs4874084	A	G	1	1	1
8	13001846	rs532841	C	T	0.5	0.5	0.5
9	18940787	rs6475273	G	A	0.7	0.7	0.7
9	130386039	rs10760566	A	C	1	1	1
10	5129685	rs12387	G	A	0.8	0.8	0.8
10	91152477	rs303211	A	G	1	1	1
10	129790848	rs3191122	C	T	0.2	0.2	0.2
11	6579290	rs2288283	G	A	1	1	1
11	603208	rs1131665	T	C	0.3	0.3	0.2
11	10783994	rs11042964	T	C	0.4	0.4	0.2
11	27636238	rs2353512	T	C	1	1	1
11	77411857	rs11237362	T	C	0.6	0.6	0.6
11	36059804	rs1138807	A	G	0.3	0.3	0.2
12	47542065	rs1565933	G	C	0.9	0.9	0.9
12	12387353	rs3741798	C	T	0.8	0.8	0.8
12	67011218	rs3741808	G	T	0.6	0.6	0.5
13	24377541	rs17402892	A	C	0.3	0.3	0.3
13	30129806	rs7984952	T	C	0.6	0.6	0.6
13	31674603	rs2428249	T	C	0.5	0.5	0.5
14	22444275	rs2295680	G	A	0.8	0.8	0.8

Chr	Position (hg18)	dbSNP (build 132)	Major Allele	Minor Allele	Sequenom MAF	SAMtools MAF	SPLINTER MAF
14	22899882	rs2231798	T	C	0.2	0.2	0.3
14	22944347	rs2277473	G	T	0.3	0.3	0.3
15	88027951	rs7169981	C	A	1	1	1
15	70854287	rs11634630	T	C	1	1	1
15	80361934	rs11630197	T	C	0.3	0.3	0.2
15	98927825	rs2411837	C	G	0.9	0.9	0.8
16	14241627	rs75963814	C	A	0.8	0.8	0.8
16	19950831	rs2147865	T	C	0.4	0.4	0.4
16	5037948	rs1127920	A	G	0.8	0.8	NC
16	73885417	rs2073619	A	G	1	1	1
16	2087164	novel	G	T	0.6	0.5	0.3
16	27659636	rs12447120	C	T	0.3	0.2	0.3
16	49302700	rs2066843	C	T	0.7	0.7	0.7
16	69453534	rs1626593	C	T	0.5	0.5	0.4
17	10344771	rs17811250	G	A	0.5	0.5	0.5
17	29977371	rs4795954	G	A	1	0.6	0.7
17	30792312	rs1822403	G	A	0.5	0.5	0.4
17	3065758	rs4325604	T	C	1	1	1
17	71773272	rs2585751	T	C	0.6	0.6	NC
17	3271560	rs227787	A	G	0.5	0.5	0.5
17	75701704	rs1800310	A	G	0.4	0.4	0.5
17	3271005	rs56379678	C	T	0.2	0.2	NC
18	31948118	rs1050631	G	A	0.3	0.3	0.3
18	41573517	rs1058396	G	A	0.5	0.5	0.5
18	46065372	rs17660776	G	A	0.3	0.3	0.3
18	72740115	rs608433	A	G	1	1	1
18	74854576	rs7240860	A	G	0.8	0.7	0.7
19	62494636	rs2041110	G	A	0.8	0.8	0.7
19	6870624	rs373533	A	C	1	1	1
19	40346991	rs1672994	T	C	0.3	0.3	0.2
19	44889764	rs4830	T	C	1	1	1
19	45902896	rs3865452	T	C	0.8	0.7	0.8
19	53293266	rs251684	T	C	1	1	1
19	7604320	rs1862514	C	T	0.8	0.8	0.8
19	40242718	rs45512696	C	T	0.4	0.4	0.5
19	49192318	rs398235	A	T	1	1	1

Chr	Position (hg18)	dbSNP (build 132)	Major Allele	Minor Allele	Sequenom MAF	SAMtools MAF	SPLINTER MAF
19	62494618	rs2041109	C	T	0.8	0.8	0.8
20	19209623	rs1569767	G	A	1	1	1
20	31445510	rs291700	T	C	0.9	0.9	0.9
20	36372466	rs5743507	G	C	0.4	0.4	0.3
20	60346522	rs8124907	A	G	0.3	0.3	0.2
21	42859024	rs228104	G	A	0.7	0.7	0.7
21	42736590	rs868092	A	G	0.4	0.4	0.5
21	39564059	rs6517532	C	T	0.7	0.7	NC
21	42286800	rs915837	C	T	1	1	1
21	42725301	rs13052676	C	T	0.3	0.3	0.2
22	35792872	rs2235321	G	A	0.4	0.4	0.3
22	38747726	rs5995793	C	T	0.8	0.8	0.9
X	153234908	novel	G	A	0.4*	0.4*	0.4*
X	15242479	rs5935944	A	G	0.9*	0.9*	0.9*
X	70260346	rs12841977	A	G	0.2*	0.2*	NC
X	135254359	rs4829829	A	G	0.8*	0.8*	0.8*
X	148774553	novel	A	G	0.8*	0.8*	0.8*
X	2642482	rs17849631	C	T	0.5*	0.5*	0.5*
X	151054049	rs210586	C	T	0.9*	0.9*	0.9*

Additional Table 3. Individually genotyped variant positions from pooled exome sequencing. The asterisk (*) denotes that in this pool of 4 females and 1 male, we assumed nine X-chromosomes and the minor allele frequencies are rounded accordingly. There were a total of 127 variant positions not-included on the genome-wide array that were individually validated by individual genotyping using Sequenom MassArray. Six of these positions were novel variants not found in dbSNP 132. NC = no call made by the SPLINTER algorithm.

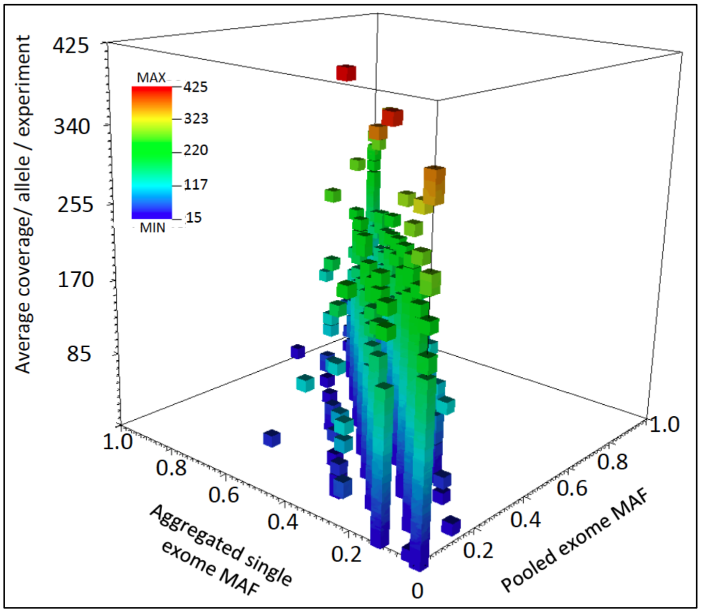

Additional Figure 3. Discordance between single and pooled exome minor allele frequency estimates is correlated to a lack of sequencing coverage. The 2,937 variant positions analyzed in Figure 1 were graphed against the relative coverage per variant position (vertical axis). The broader base of the plot (dark blue cubes) demonstrates that outliers are most likely due to a lack of sequencing coverage in either the individual or pooled sequencing rather than the introduction of systemic false positive artifacts.

A2M	C4orf34	CTNNA3	FABP2	HK3	LMX1B	NR1D1	PPARD	SMAD1	TUSC3
ABCA1	C9orf11	CTNND2	FABP3	HLA-DRB1	LRP1	NR1H3	PPARG	SMAD2	UBE2A
ABHD16A	C9orf3	CYP1A1	FABP4	HNF1A	LRP1B	NR1H4	PPARGC1A	SMAD3	UBE2G2
ACADM	CADM1	CYP3A5	FABP5	HNF1B	LRPPRC	NR2F1	PPARGC1B	SMAD5	UBE2H
ACADVL	CADPS2	DAB2	FABP7	HRAS	LY86-AS1	NR2F2	PPP2R2B	SMARCE1	UCP2
ACCN1	CALHM1	DCAF4L1	FADS1	HS3ST3B1	LYST	NR3C1	PPTC7	SOCS2	ULK1
ACE	CAMTA1	DCPS	FADS2	HSF1	LZTR1	NR4A2	PRDM16	SOD1	VASH1
ACOT1	CARD14	DDB1	FADS3	HSPA9	MAFA	NRF1	PREX1	SOD2	VPS13D
ACOX1	CAT	DDIT3	FAHD1	HSPB1	MAFF	NT5DC1	PRKAA2	SORCS1	VPS72
ADAMTSL1	CD36	DEFB1	FAS	HSPC159	MAPK8	OBFC1	PRNP	SORCS2	WDR72
ADARB1	CDK14	DGAT1	FBXO32	HTT	MAPK9	OGDH	PROM1	SORL1	WEE1
ADARB2	CDK6	DGAT2	FGFR1	IDE	MAPT	ONECUT1	PROP1	SOX18	WNT5B
ADIPOQ	CDKN2A	DKC1	FHIT	IDH3A	MB	OR56A1	PROX1	SOX9	WRN
ADRB2	CEACAM16	DKK1	FOS	IDH3G	MDH2	PAK1	PTEN	SREBF1	WWC1
AHR	CEBPB	DLGAP5	FOXA1	IGF1	MEF2A	PAPD5	PTK2	SREBF2	WWOX
AIF1	CETP	DLST	FOXA2	IGF1R	MEF2C	PAPPA2	PYGO2	SSPN	XDH
AKT1	CGNL1	DMRT1	FOXA3	IGF2	MEF2D	PARK2	RAB9A	STAT1	XKR6
AKT2	CHRM2	DNMT3a	FOXC1	IL10	MEOX2	PAX2	RAD21	STUB1	XRCC5
AKT3	CHST11	DNMT3b	FOXC2	IL12A	METAP1	PAX3	RAD51L1	SUMF1	YBEY
ALAD	CIDEA	DOCK2	FOXK1	IL1A	METTL20	PAX7	RAD51L3	SYNE1	ZAK
ANGEL1	CLK2	DOCK8	FOXO1	IL1B	MFN2	PCK2	RAE1	SYT13	ZBTB20
APBB2	CLPTM1L	DPT	FOXO3	IL6	MINPP1	PCNA	RNF145	TADA2A	ZNF562
APOA4	CLU	DRD2	FOXO4	INS	MIR181A2	PCNXL2	RPS6KB1	TAPBPL	ZWINT
APOC1	CLYBL	DSG1	FST	INSR	MKL1	PDHB	RPS6KB2	TBL1XR1	
APOC3	CNR1	DTNBP1	FTO	IRF6	MMP10	PDK4	RRP12	TBXAS1	
APOE	CNR2	DYNLL1	FUS	IRS1	MMP7	PFKFB3	RUNDC1	TEK	
ARHGAP1	CNTN5	DYNLL2	GAB2	IRS2	MNAT1	PGAM1	RXRA	TERC	
ARNTL	COMMD4	DZIP3	GATA4	ITGA1	MOCS1	PGAM2	S100B	TERF1	
ATG2B	COMP	EBF3	GBA3	ITSN1	MSRA	PGK1	SDCBP2	TERF2	
ATM	COMT	EEF1A1	GCLM	JUN	MTHFR	PGPEP1	SDHC	TERT	
ATP2B4	COQ7	EFEMP1	GH1	KAT2A	MTOR	PGR	SEMA6A	TF	
ATP5O	CPT1A	EFTUD1	GHR	KCTD1	MTTP	PHYHIP	SERINC3	TFAM	
ATR	CPT1B	EIF4E3	GIP	KIAA0020	MYBBP1A	PICALM	SH2D4A	THRB	
AVPI1	CPT2	EIF5	GK	KIAA0174	MYC	PIK3C3	SH3GLB1	TMEM2	
BACE1	CR1	ELAVL1	GPC6	KIAA1377	MYF5	PIK3CB	SH3RF2	TMTC3	
BCHE	CREB1	ELF2	GRIN2B	KIF13B	MYF6	PIK3IP1	SHC1	TNF	
BDNF	CREBBP	ELL2	GRN	KL	MYO9B	PITPNM3	SIK1	TNFRSF11A	
BHLHE40	CREBZF	ENO1	GSK3B	KLRF1	MYOD1	PLAU	SIRT1	TNFSF8	
BIN1	CRTC1	ENOX1	GSTM1	LARGE	MYT1L	PLEKHA1	SIRT2	TOMM40	
BRCA1	CRTC2	EP300	GSTP1	LASS3	NAV2	PLEKHA7	SIRT3	TOX	
BRE	CSF1R	EPB41L4B	GSTT1	LBH	NBEA	PLEKHG3	SIRT4	TP53	
BTG3	CSMD3	ERCC2	H2AFX	LDHB	NCOR1	PLK3	SIRT5	TP63	
BTNL2	CSNK1G2	ERCC8	H6PD	LDLR	NDUFA11	PMP2	SIRT6	TRIM25	
BUB1B	CST3	ERGIC1	HADHA	LEF1	NFKB1	POLG	SIRT7	TRRAP	
BUB3	CTBP1	ESR1	HADHB	LINGO2	NFKBIE	PON1	SLC25A21	TSC22D1	
C10orf2	CTBP2	ESRRA	HDDC2	LIPC	NFKBIZ	POT1	SLC4A4	TSHB	
C1orf151	CTDSP2	ESRRG	HELLS	LLGL1	NOTCH1	POU1F1	SLC6A3	TSHR	
C2	CTNNA1	EXOSC6	HK1	LLPH	NPM1	POU2F1	SLC6A4	TTC27	
C20orf79	CTNNA2	FABP1	HK2	LMNA	NR0B1	POU5F1	SLC6A7	TUBB	

Todd Druley 11/7/12 9:23 AM

Deleted:

A2M	C4orf34	CTNNA3
ABCA1	C9orf11	CTNND2
ABHD16A	C9orf3	CYP1A1
ACADM	CADM1	CYP3A5
ACADVL	CADPS2	DAB2
ACCN1	CALHM1	DCAF4L1
ACE	CAMTA1	DCPS
ACOT1	CARD14	DDB1
ACOX1	CAT	DDIT3
ADAMTSL1	CD36	DEFB1
ADARB1	CDK14	DGAT1
ADARB2	CDK6	DGAT2
ADIPOQ	CDKN2A	DKC1
ADRB2	CEACAM16	DKK1
AHR	CEBPB	DLGAP5
AIF1	CETP	DLST
AKT1	CGNL1	DMRT1
AKT2	CHRM2	DNMT3a
AKT3	CHST11	DNMT3b
ALAD	CIDEA	DOCK2
ANGEL1	CLK2	DOCK8
APBB2	CLPTM1L	DPT
APOA4	CLU	DRD2
APOC1	CLYBL	DSG1
APOC3	CNR1	DTNBP1
APOE	CNR2	DYNLL1
ARHGAP1	CNTN5	DYNLL2
ARNTL	COMMD4	DZIP3
ATG2B	COMP	EBF3
ATM	COMT	EEF1A1
ATP2B4	COQ7	EFEMP1
ATP5O	CPT1A	EFTUD1
ATR	CPT1B	EIF4E3
AVPI1	CPT2	EIF5
BACE1	CR1	ELAVL1
BCHE	CREB1	ELF2
BDNF	CREBBP	ELL2
BHLHE40	CREBZF	ENO1
BIN1	CRTC1	ENOX1
BRCA1	CRTC2	EP300
BRE	CSF1R	EPB41L4B
BTG3	CSMD3	ERCC2
BTNL2	CSNK1G2	ERCC8
BUB1B	CST3	ERGIC1
BUB3	CTBP1	ESR1
C10orf2	CTBP2	ESRRA
C1orf151	CTDSP2	ESRRG
C2	CTNNA1	EXOSC6
C20orf79	CTNNA2	FABP1

Unknown

Formatted: Font:Times New Roman

Additional Table 4. Candidate genes chosen for resequencing from the Long Life Family Study (LLFS). These 464 genes were chosen by LLFS investigators for resequencing based on published data relevant to the study aims.

Todd Druley 11/7/12 9:24 AM
Formatted: No underline

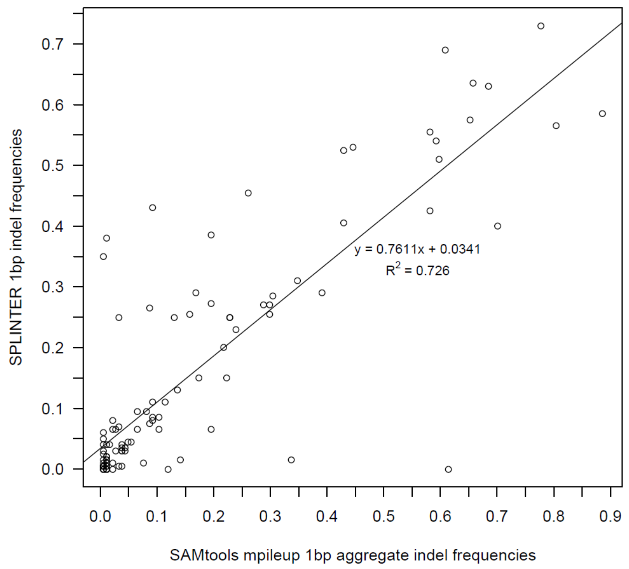

Additional Figure 4. Concordance in INDEL identification between pooled custom capture sequencing analysis by SPLINTER and SAMtools from the same indexed individuals. Of the 110 INDELS called with ≥20-fold coverage in individual analysis, we identified 99 (90%) with SPLINTER. For rare INDEL calling, 54 of the 110 were found at a frequency of <5%, and SPLINTER detected 45 (83.3%).

Additional Table 5.

Blockers for hybridization:

5′-CAAGCAGAAGACGGCATACGAGAT**IIIIIII**GTGACTGGAGTTCAGACGTGTGCTCTTCCGATCTp-3′
5′-AGATCGGAAGAGCACACGTCTGAACTCCAGTCAC**IIIIIII**TCTCGTATGCCGTCTTCTGCTTGp-3′

IIIIIII = 7bp stretch of deoxyinosine residues

Adapters (annealed together to form Y-shaped adapter):

5′-AGACGGCATACGAGAT**NNNNNNN**GTGACTGGAGTTCAGACGTGTGCTCTTCCGATCT-3′
5′-pGATCGGAAGAGCGTCGTGTAGGGAAAGAGTGTAGATCTCGGTGGTCGCCGTATCATT-3′

NNNNNNN = 7bp index

Pre-Hybridization PCR primers:

5′-CAAGCAGAAGACGGCATACGAGAT-3′
5′-AATGATACGGCGACCACCGAGATC-3′

Post-Hybridization PCR primers:

5′-CAAGCAGAAGACGGCATACGAGAT-3′
5' AATGATACGGCGACCACCGAGATCTACACTCTTTCCCTACACGACGCTCTTCCGATCT-3′

These are the 96 indices used and integrated into the adapter sequence:

ACGCCTC	AACTATC	ATGAATT	GTGACAG	AGTTAAA	ATTCACC	CCCCTGC	GAGCAGA
CAAATAC	AGAACAT	AGTATCT	TACGGCC	CGTACTC	TTTTTAC	GCTGGGA	TTATACC
TATCTGT	GATATTG	GATTAGG	ACGTGTG	TGCATTT	TCGGGAG	TGTTTGA	CTACTCT
CCCGCGT	AAACGGC	TTCAGGT	CCGGACG	GGACTCC	TTACCTA	TCTGAAA	GAAGTGT
AGCGACG	ATCTCGA	ATCCAGT	CTTCCAC	CCATGTA	CTAAATG	TCTTGCC	ACCAAGG
TGAGAAC	TCCCGGA	GGCCATC	CATGATC	ATGGGCA	ACTTTCG	TTCGCCT	GTCGGAG
AAGGTTT	GGGAGGC	GTCTGTA	TACTACA	GTATGGT	CATAACT	CGGGGGG	AGGAAGA
GCGTATT	CGGCTTG	CGGCGCT	GCATCTC	TGTAGCG	TTGACCC	CAGCGAA	AGCACTG
TATGCCA	GCGTTGC	GGGTGAG	GTTCTTC	AGTGGGT	GTAATCA	CGCACGA	GCGCTAG
GGTCCGG	CCCAGCA	CGATCAC	AATACTA	GCCGTAC	CTGTGTC	TGCTGAT	TGGTCTC
GGGGCAA	AGGTCCT	ACCATTC	TCCATAG	TTGGAGT	CCGAAAA	GTGGCTT	ATCGTAA
TGGCACA	GACTCTG	TATGGTT	TTAATTC	CTCCGTT	AGGATAC	CTTCGCG	CAATAGC

Certain indexes were not used in the multiplexes of 22-24 (yellow) or 30-32 (turquoise). Indexes were designed with a Hamming distance of 2 bp to allow identification despite a 1 bp sequencing error. The reverse compliment of these sequences is what was actually seen in the raw sequence data and analyzed.

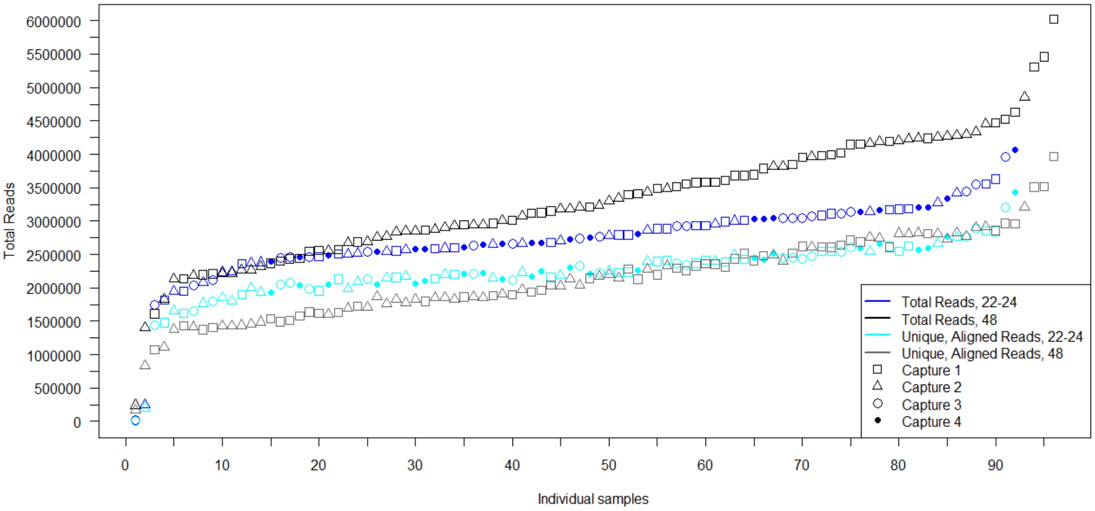

Multiplex size(s)	22-24	48
Color on graph	Dark/light blue	Black/gray
Total number of individuals/ lane of sequencing	92	96
Total reads (millions)	129.8	162.5
Perfect index match (%)	94.8	94.9
1 bp mismatched index (%)	1.9	2.8
Total indexed reads (%)	96.7	97.7
Fold difference in raw data between individuals (% of individuals included)	2.30 (90)	2.83 (96)
Raw read counts/pp (millions)	1.75 - 4.06	2.13 - 6.03

Additional Figure 5. Raw and aligned read uniformity. The raw and aligned, unique read counts per individual in four hybridizations of 22-24 individuals (Additional Table 1, rows 7-10) and two hybridizations of 48 individuals (Additional Table 1, rows 14-15). Individuals are ranked from lowest total read count to highest total read count by total reads per person (black and dark blue symbols). Dark and light blue symbols: the open squares, circles, triangles, and closed circles indicate the four different captures of 22-24 individuals. Black and gray symbols: the open squares and circles indicate the two captures of 48 individuals each. The distribution of symbols across each dataset demonstrates that individual results did not cluster within captures. The table lists the metrics for each lane of sequencing, the fold difference between individuals within each lane and the percentage of indexes identifiable within each raw data set.

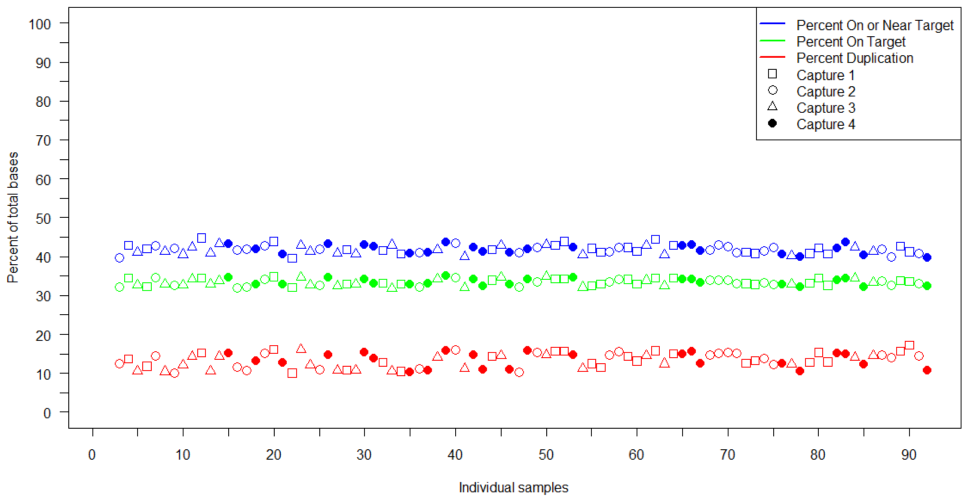

Additional Figure 6. PCR duplicates and capture efficiency for multiplexes of 22-24 (Additional Table 1, rows 7-10). The graph shows the individual percent of: PCR duplicates (red symbols), aligned data on target (green symbols) and aligned data on and near targeted intervals (blue symbols). The two samples with very low read counts are not shown and the rest of the samples (n=90) are ranked from lowest total read count to highest total read count. Different shapes and filling indicate the four independent captures that were combined for sequencing. "On and near target" is defined as bases that align on or within 250 bp of target intervals after removing duplicates. "On target" is defined as bases that align directly on targeted intervals after removing duplicates. The "Individual samples" numbers correspond to the ones used in Additional Figure 3.

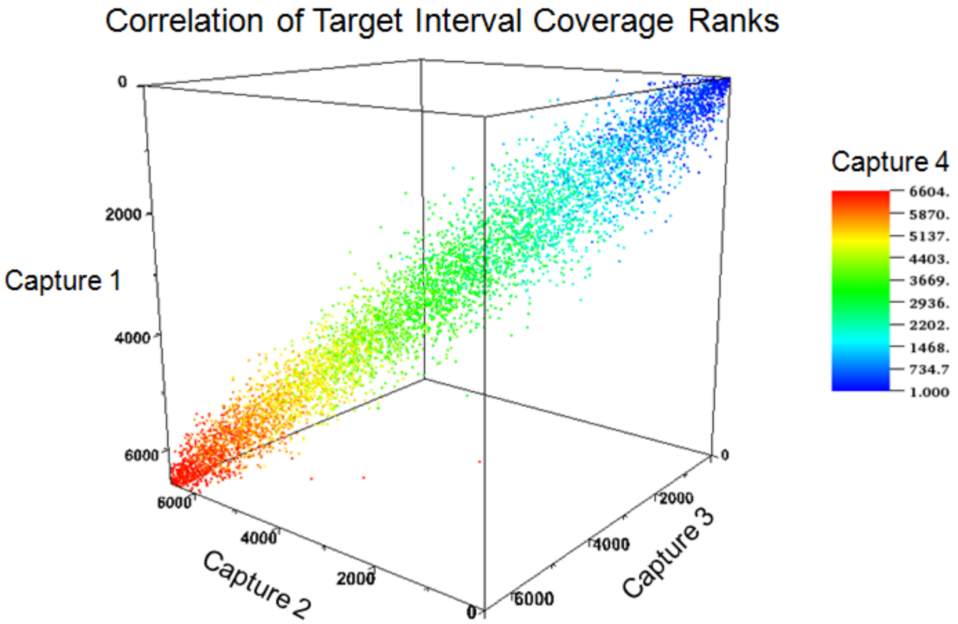

Additional Figure 7. Targeted intervals ranked by coverage achieved between different samples and captures. One sample from each of the four multiplexes of 22-24 was chosen for this plot. Observed indexes ACAGATA, ATCAGCA, TGCTGGG, TTACGAT where chosen, with read counts ranging from (1,580,586 - 1,599,797). Of the 6,966 distinct targeted intervals, the 6,604 that achieved greater than 0x average coverage in all 4 of these samples were considered for this plot. All intervals were ranked by average coverage within each sample with 1 being the highest and 6,604 being the lowest and plotted. Between all four samples, the average pairwise R^2 = 0.936, showing a high level of uniformity between captures. To determine if candidate regions were enriched uniformly across the four multiplexes, we ranked the 6,966 intervals that were covered by baits by coverage in one sample from each of the four captures. Comparing the ranks of all 6,604 intervals with over >0X coverage in each of the four samples, the average pairwise R^2 = 0.936 (Additional Figure 4). Thus, our method performs uniformly with respect to both aggregate metrics of an entire capture as well as bait-to-bait capture efficiency.

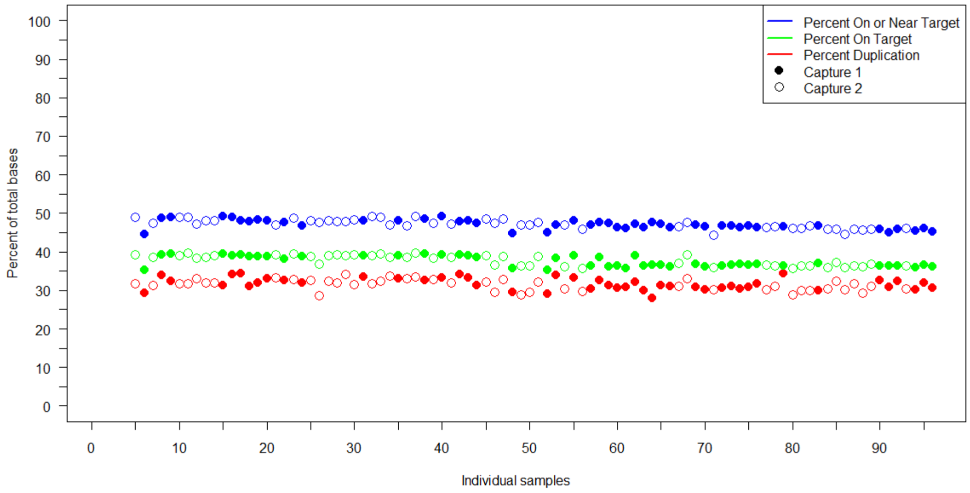

Additional Figure 8. PCR duplicates and capture efficiency for multiplexes of 48 (Additional Table 1, rows 14-15). The graph shows the individual percent of: PCR duplicates (red symbols), aligned data on target (green symbols) and aligned data on and near targeted intervals (blue symbols). The two samples with very low read counts are not shown and the rest of the samples (n=90) are ranked from lowest total read count to highest total read count. Different shapes indicate the two independent captures that were combined for sequencing. "On and near target" is defined as bases that align on or within 250 bp of target intervals after removing duplicates. "On target" is defined as bases that align directly on targeted intervals after removing duplicates. The "Individual samples" numbers correspond to the ones used in Additional Figure 3. We see an improvement in overall on/near target percentage compared to our smaller multiplexing experiments due to improving our blocking strategy by adding 1 uL of the 58 bp post-hybridization PCR primer Illumina PE 1.0 (the longer oligo listed under Post-hybridization PCR primers on Additional Table 5) to the Hybridization Capture step outlined in the Supplemental Methods.

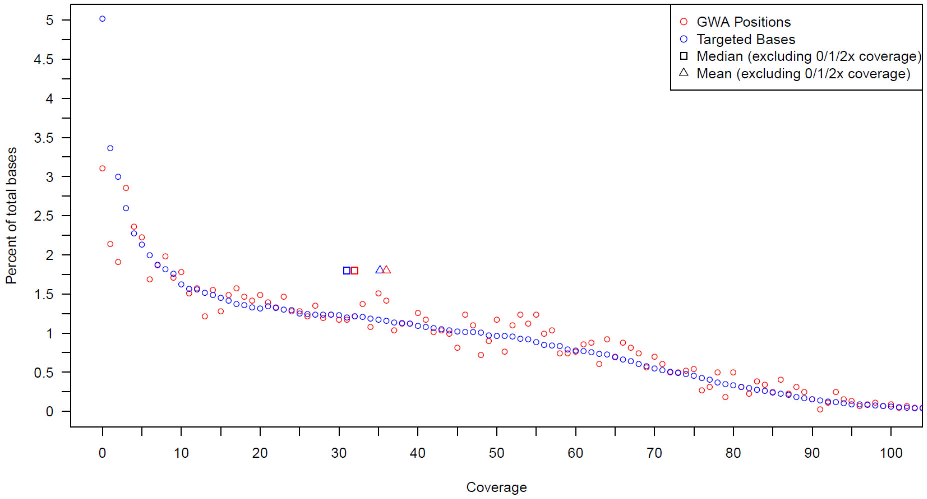

Additional Figure 9. Comparing genome-wide array position coverage to sequencing coverage. This plot was generated using the observed index TAGTATT from the set of 22-24 multiplexed captures, which achieved 1,404,000 million reads, closest to the average for all 92 samples. Blue circles: percent of targeted bases at specified coverage threshold. Red circles: percent of array positions at specified coverage thresholds. Squares: median coverage achieved when considering only positions at 3-fold coverage or greater. Triangles: mean coverage achieved when considering only positions at 3-fold coverage or greater. The close overlap of the mean and median indicate the array-based positions accurately reflect the sequencing positions with respect to the overall coverage achieved.

Transition : Transversion (Ti/Tv) ratio analysis

In addition, we analyzed the transition:transversion (Ti/Tv) ratio of all variant calls in our 22-24 individual multiplexes according to DePristo [25]. We expect known variants to have a Ti/Tv ratio ~3.3 and novel variants to have a Ti/Tv ratio ~2.8-3.0 within coding regions. We considered all SNV calls with at least 5-fold and 10-fold coverage, and found the Ti/Tv ratio was 3.30 and 3.34 respectively, supporting the conclusion that we are detecting variants accurately. When considering novel, non-SVM variant calls with any level of coverage, there were 1,235 calls made that yielded a poor Ti/Tv ratio of 0.70. However, by implementing coverage thresholds of 10-fold and 15-fold coverage, the Ti/Tv ratio improved to 1.52 and 2.41, respectively. This suggests that novel variants with less than 15-fold coverage are more likely to be false positives.

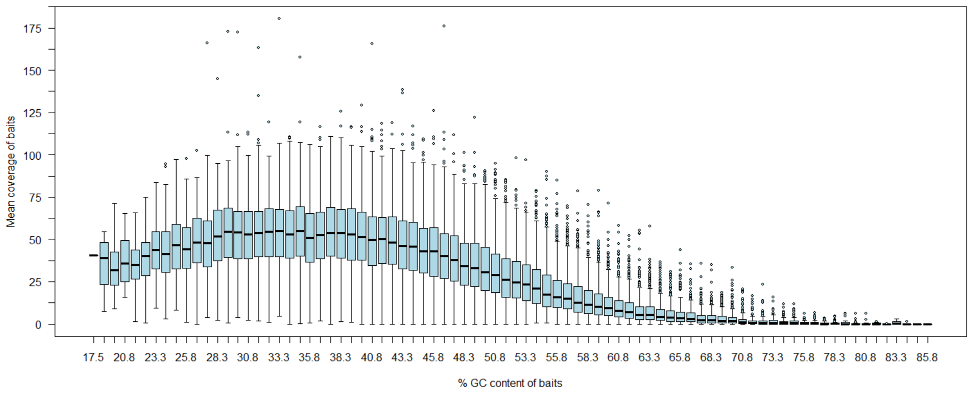

Additional Figure 10A: Coverage achieved depends on GC content of designed baits. This plot was generated using the observed index TAGTATT from the set of 22-24 multiplexed captures, which achieved 1,404,000 million reads, closest to the average for all 92 samples. The average coverage for each bait was plotted against the % GC content of the bait with the boxplot function in R.

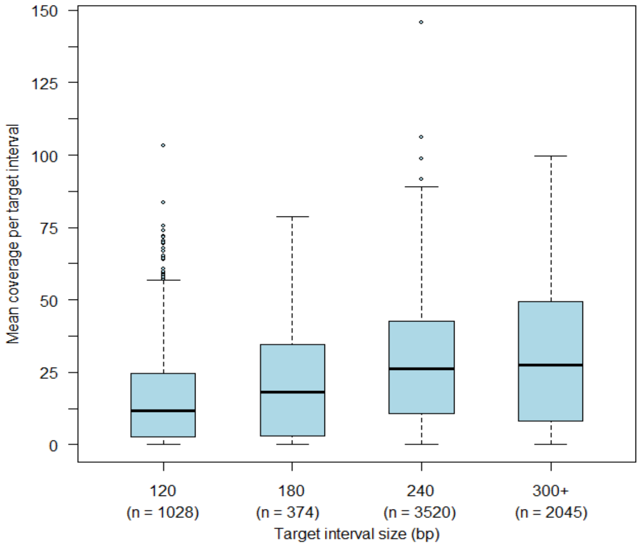

Additional Figure 10B: Coverage achieved depends on target interval size. This plot was also generated using the observed index TAGTATT. The average coverage for each bait was plotted against the target interval size that the bait belonged to with the boxplot function in R. Higher coverage in targeted intervals ≥240 bp was observed relative to 120 bp and 180 bp intervals. While partially due to having a greater number of bases targeted by two baits in these intervals due to specifying 2X tiling frequency, we feel this is more largely a result of flanking sequence from a fragment for one bait yielding coverage for an adjacent bait.

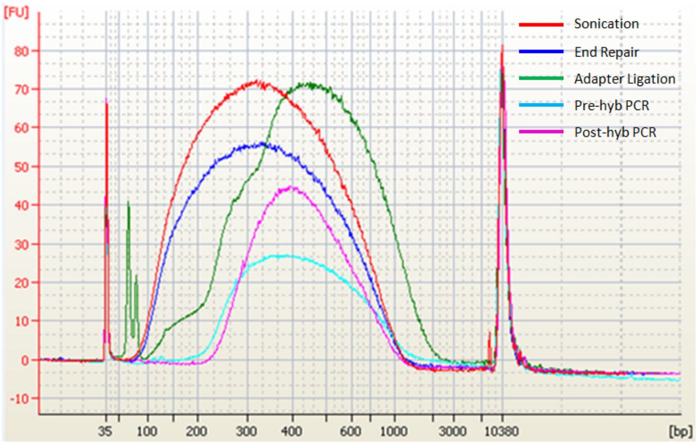

Additional Figure 11: Bioanalyzer traces of subsequent steps during indexed library preparation. Red line: individual sonicated and purified sample. Blue line: individual end-repaired and purified sample. Green line: individual adapter ligated and purified sample. The three different populations beyond 100bp may indicate fragments with 0, 1, and 2 adapters ligated. The bimodal peaks <100 bp correspond to unligated adapters. Cyan line: pre-capture, purified PCR product. Magenta line: post-capture, purified PCR product. The fact that the majority of our fragment sizes were >200 bp supports our previous conclusion that our fragments of ≥240 bp demonstrated higher total coverage due to flanking sequence from one bait contributing to total coverage for an adjacent bait (see Additional Figure 10B).

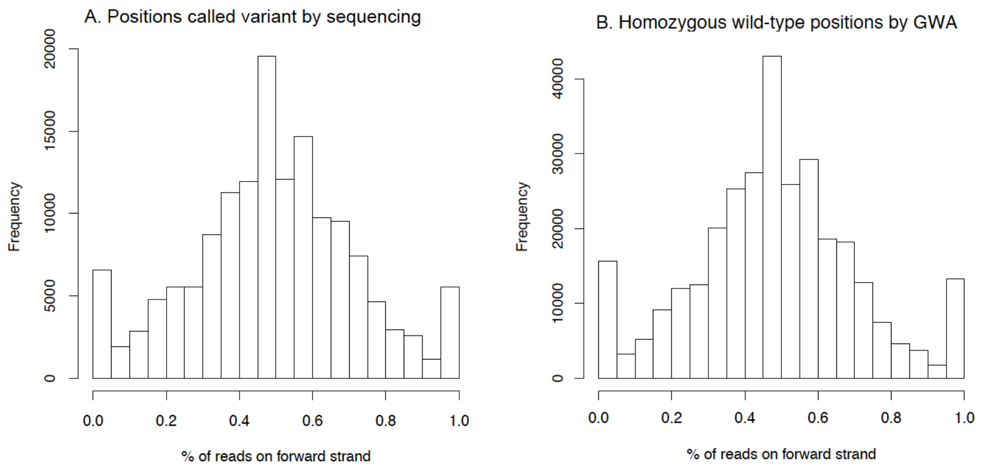

Additional Figure 12. Strand bias is not adversely affecting sequencing calls. To explore whether strand bias in sequencing output was adversely affecting variant calls in the custom capture sequencing from the multiplexes of 48 individuals (Additional Table 1, rows 14-15), we summed all variant calls from all 96 individuals and compared the total number of positions (Y-axis) against the percentage of raw sequencing reads generated from the forward strand (X-axis). Each column represents a "bin" of a given number of variants with a given percentage of raw reads generated from the forward strand. We performed this comparison for all called variants, known and novel, at all MAFs (Panel A; n = 148,972), against the gold standard dataset of all positions called homozygous wild-type by GWA (Panel B; n = 308,842). The two profiles are very similar and the central peak indicates that the majority of positions had similar amounts of raw data from each strand, suggesting that strand bias is not adversely affecting sequencing calls for all positions not called homozygous wild-type by GWA.

Todd Druley 11/7/12 9:24 AM
Formatted: No underline

	Multiplexes of 48				Multiplexes of 30-32			
Variant alleles in pool	Array positions	Reads matching wild type allele	Reads matching variant allele	% of reads matching variant allele	Array positions	Reads matching wild type allele	Reads matching variant allele	% of reads matching variant allele
0	1575	5323207	7690	0.14	1693	5334506	6722	0.13
1-9	593	1887301	5194	0.27	560	1595827	2028	0.13
10-19	254	723551	6060	0.83	312	750413	2491	0.33
20-29	171	400847	5732	1.41	208	454931	2485	0.54
30-49	287	555231	12194	2.15	279	513783	4297	0.83
50-69	193	293174	10222	3.37	201	272377	3476	1.26
70-89	121	111113	5090	4.38	147	138884	2274	1.61
90-109	108	66798	3801	5.38	122	76696	1554	1.99
110-129	86	37334	2637	6.6	86	29456	725	2.4
130-149	99	19368	1686	8.01	90	18005	534	2.88
150-169	57	4778	456	8.71	71	4520	168	3.58
170-188	110	637	59	8.48	98	119	7	5.56

Additional Table 6: The percentage of misattributed indexes is improved by an additional purification of unligated adapter sequences. To test whether unligated adapters were the source of misattributed indexes in the multiplexes of 48 (Additional Table 1, rows 14-15), we implemented an additional purification with 9.45% PEG and 1.25M NaCl after pooling the adapter-ligated samples and prior to the subsequent PCR enrichment to more thoroughly remove any unligated adapters in the multiplexes of 30-32 (Additional Table 1, rows 11-13). We focused on positions with a MAF around 50% (range 39-59%), which provides more positions to query (215,580 reads mapped to wild type alleles, 3,828 reads mapped to variant alleles) where multiple individuals should possess one of three distinct genotypes (AA, AB, BB). Of the 96 individuals captured in two multiplexes of 48, 94 had valid array genotyping. All positions with 188 valid allele calls were binned according to the number of GWA-called variant alleles in the cohort. Of the 92 individuals captured in three multiplexes of 30-32, all 92 had valid array genotyping. Analysis was identical to the multiplexes of 48, with two samples excluded due to low coverage and one sample excluded due to having a very high mismatch rate, possibly due to genotyping performing poorly or a sample handling mishap not indicative of the method as a whole. Introducing the extra purification step improved the percentage of misattributed indexes. With the additional purification in place, we find an average of 1.8% of variant reads that are attributed to an individual who was homozygous wild type by array. At base positions where no variant alleles were called by array, 0.13% of reads erroneously contain a sequence variant, likely due to sequencing error or alignment artifacts. Subtracting this background from the 1.8% of misattributed indexes at positions with a validated variant in approximately half of all alleles yields 1.67%. We assume that this index switching is a random and stochastic process, suggesting that we only “see” half of reads with an inappropriate index because the other half would adjoin with a read having a matching genotype (e.g. instead of an index switching from a wild type read to a variant read, it switches from a wild type read to another wild type read). Thus, we conclude that with the additional purification a total of ~3.4% of reads contain misattributed indexes.

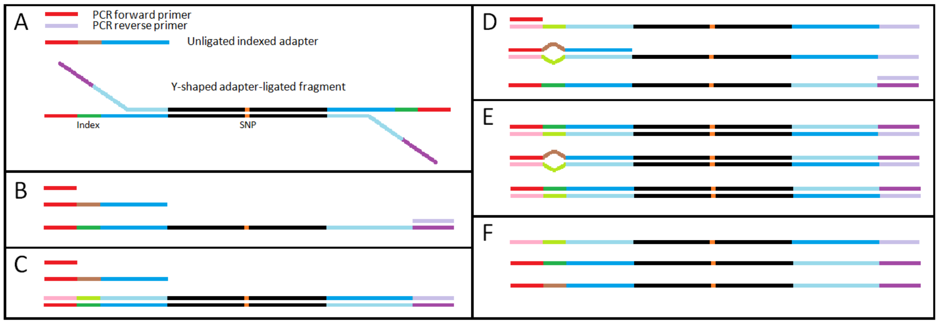

Additional Figure 13: Schematic diagram demonstrating how indexed adapters may become affixed to a different source molecule. A) Primers, Y-shaped adapter-ligated fragments, and unindexed ligated adapters are mixed together prior to PCR amplification. B) After denaturation during the first PCR cycle, the reverse primer sits down on its complementary site during the annealing step. The non-indexed strand of the unligated Y-shaped adapter is not shown. C) During the extension phase of the first PCR cycle, the reverse complement strand of the template is synthesized. D) After denaturation during the second PCR cycle, the primers sit down on their complementary site during the annealing step. Additionally, any unligated adapter can sit down and serve as a primer. An indexing strand with a different index is shown. E) During the extension phase of the second PCR cycle, any individual-specific sequencing alterations can be attributed to incorrect indexes. F) After the second PCR cycle, the strand attributed to the wrong index can be amplified exponentially.

Supplemental Methods

We are starting with 70-375ng of purified genomic DNA per person in the protocol, suspended in 15ul of TE. [Note: For multiplexes of 48, we have started with 350 ng, 280 ng, 210 ng, 140 ng, and 70 ng of DNA/person and achieved equivalent results in terms of percent duplication, data on target, coverage achieved, sensitivity, and specificity (data not shown).]

Indexed Sequencing Library Preparation Protocol for Hybridization Capture

Prepare Y-shaped adapters:

Indexed oligos and the non-indexed oligo (Additional Table 2) were mixed and diluted in TE to a final concentration of 5uM. Sodium chloride was added in to a final concentration of 50mM. Each indexed solution was heated in a thermocycler to 95C for five minutes and then cooled by 1C in 30 second intervals to a final temperature of 10C.

Sonication:

1. For each sample to be sonicated, mix 50 ul Ampure XP bead solution with 100 ul water and add mixture to corresponding sample well of a PCR plate for sonication (we use Thermo-Fast 96 PCR Plate, cat #: AB-1900).
2. Place the plate on plate magnet (Invitrogen, DynaMag -96 side skirted, cat # 120.27), let beads pellet, and discard all solution. Only the physical beads should remain in the wells.
3. Add 97 ul 1x TE to each well of the plate to be sonicated. **Note:** It is recommended to sonicate in TE instead of water to minimize formation of single-stranded DNA.
4. Add 45 ul of 5M NaCl to each well of the sonication plate to be sonicated.
5. Add 15 ul of DNA sample to each well of the sonication plate to be sonicated. **Note:** Our DNA was concentrated to 22-25 ng/ul for this experiment. **Adjustment:** We have since shown we can get equivalent results using as little as 70ng input DNA per sample into the experiment.
6. Cover all wells with strip caps (Bio-Rad TCS0803). Optionally cover the top with a film cover, and/or wrap the top of the plate in parafilm to prevent sample leaks.
7. Sonicate each well in the Covaris E210 with a 7 mm vertical offset, 10% duty cycle, intensity set at 5, and 500 cycles per burst, for 10 minutes. The temperature should be below 7C at the start of the sonication. **Tip:** For best results, place weight on top of the plate to prevent floating.
8. Following sonication but prior to removing the plate from the Covaris, we recommend marking down which wells have air pockets at the bottom. Centrifuge the plate and sonicate those wells for an additional 1 minute. **Note:** We typically see less than five wells with air pockets when sonicating an entire plate.
9. Repeat step 8 twice.

This plate now has properly sonicated DNA and can be stored briefly at 4C prior to purification.

Purify and concentrate the sonicated DNA:

1. Pulse centrifuge the plate to 200 g.
2. Carefully remove the strip caps from each well to avoid cross contamination of DNA.
3. Add in 45 ul of 50% (w/v) PEG 3350 to each well.
4. Cover the plate with a film cover, being careful to seal each well. Remove the plate from the magnet and vortex vigorously for at least 10 total seconds and until there are no bead pellets in the plate. **Tip:** If bead pellets remain in the plate after vigorous vortexing, alternate the placement

of the plate within the columns of the magnet to cause the beads to move back and forth between the sides of the well. Then repeat vigorous vortexing.

5. Incubate the plate at room temperature for a minimum of 5 minutes.
6. Place the plate on a plate magnet until the beads pellet on the side (2-3 minutes). **Note:** Pulse centrifuging is not necessary as the wells are almost full and there is a continuous film of liquid throughout the tube and onto the film cover. If you are using a different plate, pulse centrifuge to 100g prior to placing on the magnet.
7. Remove the film cover and discard the supernatant by pipetting, taking care not to touch the beads with the pipette tips.
8. Add 75 ul of 70% EtOH to each well.
9. Discard the supernatant by pipetting, taking care not to touch the beads with the pipette tips.
10. Repeat steps 9 and 10.
11. Incubate the plate (25C – 37C) uncovered until ethanol droplets evaporate. **Note:** if all ethanol is removed by pipetting this should take under 5 minutes.
12. Add 10 ul of water to each well. Cover the plate with a film cover. Vortex vigorously for at least 5 seconds. Continue vortexing until the beads move from the side of the well into the water solution.
13. Incubate the plate at room temperature for 2 minutes.

Optional: Quality Control (QC) check of the DNA:

1. Pick any two wells randomly for QC purposes. Add 2 ul of water to each of these two wells. Mix the wells thoroughly.
2. Place the plate on the plate magnet.
3. For one well, reserve 2 ul of solution for Bioanalyzer 2100 (or equivalent) analysis, if necessary. This can be used to indicate the size range of the DNA after sonication and the DNA yield after sonication.
4. For the other well, use the 2 ul to measure concentration by absorbance. As long as a peak or shoulder is observed at 260 nm, regardless of the concentration, it is ok to proceed. **Tip:** Keep the plate on the magnet when removing sample to avoid any bead contamination.

This plate now has properly sonicated, purified, and concentrated DNA and can be stored at 4C prior to end repair.

Repair the ends of the sonicated, purified, and concentrated DNA:

1. Remove the resuspension buffer (RB) and end repair (ER) mix from a TruSeq kit (cat # FC-121-2001 or FC-121-2002) and allow them to thaw to room temperature. Place the ER mix on ice after thawing. **Tip:** After the RB completely thaws, prepare 1 ml aliquots to allow quicker thawing in the future.
2. Prepare a master mix of the RB and ER mix on ice. Each well will receive 2 ul of RB and 8 ul of ER mix. Make enough for excess. **Example:** For 96 wells mix 200 ul resuspension buffer with 800 ul of ER mix.
3. Centrifuge the DNA plate, remove the film cover, and place on ice.
4. Add 10 ul of the master mix to each well. **Tip:** Ensure all 10 ul is deposited in the well.
5. Cover the plate with a film cover (Thermo scientific Adhesive PCR Film, cat # AB-0558) and pulse centrifuge to 100 g, vortex briefly, and pulse centrifuge again to 100 g.
6. Incubate the plate at 30 C for 30 minutes.

This plate now has blunt-ended, sonicated DNA. We recommend proceeding with purification as soon as possible.

Purify and concentrate the blunt-ended DNA:

1. Prepare a mixture of 25% (w/v) PEG 3350 and 2.5M NaCl. **Example:** For 96 wells mix 1 ml of 50% PEG with 1 ml of 5M NaCl.
2. Pulse centrifuge the plate to 200 g. Remove the film cover.
3. Add 20 ul of this mixture to each well of the end-repaired product. **Tip:** Make sure the choice of pipette tips effectively dispense all of the PEG/NaCl solution.
4. Repeat steps 5-13 of the "Purify and concentrate the sonicated DNA" section above. **Note:** Using less than 10 ul water for elution causes us to lose uniformity in the representation of each sample in the sequence data.

Optional: QC the DNA:

1. QC the DNA as done after the previous purification, but choose two different sample wells. This will indicate the percent yield after the end repair and subsequent cleanup.

This plate now has properly blunt-ended, purified, and concentrated DNA and can be stored for an extended period of time prior to A-tailing.

Adenylate the 3' ends of the DNA:

1. Remove the RB and A-tailing (AT) mix from a TruSeq kit and allow them to thaw to room temperature. **Note:** Leave the RB at room temperature for the adapter ligation step.
2. Prepare a master mix of the RB and AT mix on ice. Each well will receive 1.67ul of RB and 8.33 ul of AT mix. Make a slight excess. **Example:** For 96 samples, mix 167ul of RB and 833 ul of AT mix.
3. Pulse centrifuge the plate to 200 g and remove the film cover.
4. Place the plate on ice and add 10 ul of the master mix to each well.
5. Cover the plate with a film cover and pulse centrifuge to 100 g.
6. Vortex the plate briefly and pulse centrifuge to 100 g.
7. Incubate the plate at 37C for 30 minutes.

After the incubation, quickly proceed to adapter ligation.

Ligate adapters to the DNA:

1. Remove the plate from the thermocycler, remove the film cover, and place the plate on ice.
2. Add 1.73 ul of resuspension buffer to each well of the DNA plate. **Tip:** Be careful to ensure all 1.73 ul gets in the wells instead of sticking to the side of the tip.
3. Cover the plate with a film cover and pulse centrifuge to 100 g. Then uncover the plate and place on ice.
4. Add 2.5 ul of pre-annealed Y-shaped adapters to their corresponding wells on the DNA plate. **Tip:** For bookkeeping purposes it is easiest to have indexes from a well in the adapter plate correspond to the same well in the sample plate.
5. Cover the plate with a film cover and pulse centrifuge to 100 g, uncover the plate, and place on ice.
6. Take out the ligation mix from a TruSeq kit and leave on ice.
7. Add 1.73 ul of ligation mix to each well of the sample plate. **Tip:** Be careful to ensure all 1.73ul gets in the wells instead of sticking to the side of the tip.
8. Cover the plate with a film cover and pulse centrifuge to 100 g, vortex briefly, and pulse centrifuge again to 100 g.

9. Incubate the sample plate at 30C for 30 minutes.
10. While incubating, take out the stop ligation buffer to thaw.
11. After the incubation, add 3.46ul of stop ligation buffer to each well. **Tip:** Be careful to ensure all 3.46ul gets in the wells instead of sticking to the side of the tip.
12. Add a film cover, vortex the plate vigorously, and spin down.

The sample plate now has adapter-ligated DNA and can be stored briefly prior to purification.

Purify and concentrate the adapter-ligated DNA:

1. Prepare a mixture of 18.64% (w/v) PEG 3350 and 2.45 M NaCl. Example: For 96 wells mix 1,140 ul of 50% (w/v) PEG 3350, 1,500 ul of 5 M NaCl, and 418 ul water together. **Tip:** If the PEG concentration is too high at this step, unligated indexed adapters will carry through and cause incorrectly assigned indexes in the sequencing output. We recommend creating a stock of PEG at 50% (w/v) and practicing this purification on sonicated commercial genomic DNA and/or a gel loading ladder to ensure the final PEG concentration at this step (ideally 9.5% (w/v)) is achieved adequately.
2. Dispense 30.58 ul of the PEG/NaCl/water mix to each well.
3. Perform steps 5-12 of the "Purify and concentrate the sonicated DNA" section from earlier in the protcol.
4. Add 20 ul of water to each well. Cover the plate with a film cover. Vortex vigorously for at least 5 seconds. Continue vortexing until the bead pellet is fully dissolved in the solution. **Tip:** We find quick pulses on the vortex are more effective than extensive vortexing with small volumes in water.
5. Incubate at room temperature for 2 minutes.
6. Transfer 17 ul of solution to a new plate for long term storage of DNA at -20C. **Tip:** Look at the tips after removing DNA to ensure beads are not transferred. If beads are on the tip, dispense the DNA back into the original plate and repeat the withdrawal.

The sample plate now has adapter-ligated purified DNA and can be stored for a long period of time prior to pooling samples.

Quantify the DNA:

We are using a fluorescent Sybr Gold (Molecular Probes) -based assay as shown in Druley et al (ref. 4). DNA concentrations should be between 4 and 35 ng/ul, depending largely on the amount of input DNA used. Check the DNA concentration of two of the highest and lowest values by absorbance methods and confirm the values follow the trends observed in the SYBR gold data. Most of the samples should cluster around similar concentrations. **Note:** It is absolutely critical to accurately quantify the DNA in this step, and we find that fluorescent quantification methods (PicoGreen, Qubit, etc.) are superior to absorbance methods.

Pool the DNA samples:

Aim to pool a total of 50 ng of DNA per individual in sets of 24-48. **Note:** If starting with 70 ng DNA, there may be some samples that do not quite meet the 50ng mark. We recommend not titrating down the amount of DNA from other samples to match the lower samples as this will increase the PCR duplication percentage throughout the entire data set. We would prefer to settle for fewer reads for a handful of samples.

Post pooling adapter removal:

1. Add 50 ul Ampure XP bead solution and 100 ul water to a DNA Lo-Bind (Eppendorf cat# 022431021) tube. Mix thoroughly.
2. Place the tube on the magnet and discard the supernatant after the beads pellet.
3. Record the volume of pooled sample. Add the sample, PEG, and NaCl to the tube such that the final concentration of PEG is 9.5% and the final concentration of NaCl is 1.25M.

Perform steps 5-12 of the "Purify and concentrate the sonicated DNA" section from earlier in the protocol, adjusting the amount of 70% ethanol added to cover the beads and eluting with 100ul of water.

Pre-hybridization PCR:

1. Add 5 ul of a mixture of 1:10 diluted pre-hybridization forward and reverse primers to the pooled DNA. **Note:** The sequences are listed in Additional Table 2.
2. Add in an equivalent total volume of the TruSeq PCR master mix. **Example:** If the pooled DNA equaled 100 ul and 5 ul of primers were added, add 105ul of PCR master mix. Note: If the amount of DNA pooled is so large that the PCR master mix becomes a limiting reagent, concentrate the sample with a standard Ampure XP purification prior to the PCR. We recommend not concentrating below 100 ul to ensure the amount of dNTPs and enzyme in the master mix do not become limiting reagents during the amplification.
3. PCR amplify the DNA with the following settings for a total of 6 cycles:
    a. 98C x 30 sec
    b. 98C x 20 sec
    c. 57C x 30 sec
    d. 72C x 30 sec
    e. 72C x 5 min
    f. 4C hold
4. When done, load 5% of the PCR product on a 2% agarose gel. A strong band should be visible predominantly between 250-600 bp. Importantly, no adapter dimer band should be observed around 120 bp.

Purify and quantify the DNA:

If no adapter dimer was observed on the gel:

1. Purify the DNA with a standard Ampure XP bead protocol and elute in 50 ul of water.
2. Quantify the DNA. **Note:** We typically see values of 50-70ng/ul of DNA after the 6 cycle PCR if pooling 50 ng of pre-hybridization DNA per individual.
3. Concentrate the sample to 147.5 ng/ul in a SpeedVac and proceed to the hybridization step.

If adapter dimer (a band at ~ 120bp) was observed on the gel:

1. Perform a 9.5% PEG 3350 (w/v) purification to remove most of the adapter-dimer. Elute in 50 ul water.
2. Quantify the DNA. **Note:** We typically see values of 50-70ng/ul of DNA after the 6 cycle PCR if pooling 50 ng of pre-hybridization DNA per individual.
3. Concentrate the sample to 147.5 ng/ul in a SpeedVac and proceed to the hybridization step.

Hybridization Capture:

1. Follow the standard Agilent indexing capture protocol (we followed "SureSelect Target Enrichment for Illumina Paired-End Multiplexed Sequencing" Version 1.2, January 2011 with a capture size under 3.0 MB) with the following exceptions:
    a. Do not add in PE index block #3.
    b. Add in 2 ul each of the extra blockers for hybridization (shown in Additional Table 2) in place of PE index block #3.

   c. Add in 1ul of Illumina PE 1.0 (the longer post-hybridization PCR primer shown in Additional Table 2).
   d. If input DNA is more concentrated than necessary, do not add in water to make up the difference as there is extra volume from the blockers.
2. Record the time the capture begins. **Tip:** Do not worry about any bubbles as they will pop quickly during the incubation at 65C. **Tip:** 5 minutes into the 24 hour incubation, quickly remove the plate and glance at the wells to see if the volumes are even and no liquid is on the sides. If liquid is on the sides, briskly and rapidly tap the plate on the bench top and put back in the incubator.

As there is some evaporation throughout the process, we routinely add the extra 3.4 ul of volume with the blockers. If this is a concern, mix the appropriate amount of sample DNA and blockers together and place on a SpeedVac until the volume is 9 ul, as specified by the standard Agilent protocol.

Post-Capture purification:

1. Follow the standard Agilent protocol. Be careful to add the DNA from the plate to the beads with wash buffer #1 as quickly as possible and to not let the solution cool down below 65C when wash buffer #2 is added.
2. After cleanup with streptavidin coated beads, perform a standard Ampure XP bead cleanup. Elute in 30 ul of water.

Post-Capture PCR:

The standard TruSeq protocol can be used almost as previously. Use:
- 14 ul input DNA
- 1.5 ul of 1:10 diluted mixture of post-hybridization PCR primers (see Additional Table 2 for sequences)
- 15.5 ul of the TruSeq PCR master mix

Alternatively, a Herculase protocol can be used:
- 14 ul input DNA
- 1.5 ul of 1:10 diluted mixture of post-hybridization PCR primers (see Additional Table 2 for sequences)
- 0.5 ul of 100 mM dNTP (25 mM each base)
- 10 ul of Herculase buffer
- 23 ul water
- 1ul Herculase

Use the same thermocycler conditions as in the “Pre-hybridization PCR” section earlier in the protocol with 14 cycles of amplification (instead of 6).

Post-Capture PCR Purification:

1. Add 50 ul Ampure XP bead solution and 100 ul water to a DNA Lo-Bind (Eppendorf cat# 022431021) tube. Mix thoroughly.
2. Place the tube on the magnet and discard the supernatant after the beads pellet.
3. **[Note:** For this and the next three steps, volumes will reflect the TruSeq PCR protocol (the left value) or the Herculase protocol (the right value).] Add all 31/50 ul of the PCR product to the tube (TruSeq/Herculase protocol).
4. Add 11.7/19.5 ul of 50% PEG 3350 (w/v) to the tube.
5. Add 15/25 ul of 5 M NaCl to the tube.

6. Add 2.3/5.5 ul water to the tube.
7. Vortex vigorously for at least 10 seconds until the beads are homogeneous within the sample.
8. Incubate for 5 minutes at room temperature.
9. Place the tube on the magnet and wait 2-3 minutes for the beads to pellet.
10. Discard the supernatant and add 500 ul of 70% EtOH.
11. Repeat step 10. **Tip:** Use a small diameter pipette tip to remove remaining liquid EtOH in the bottom of the tube.
12. Wait for the remaining traces of EtOH to evaporate by leaving the tube at room temperature or incubating at 37C.
13. Once the pellet is dry of any residual EtOH, elute the DNA in 55 ul sterile water. Vortex thoroughly for at least 5 seconds and until the beads go into solution.
14. Incubate at room temperature for 2 minutes, place tube on a magnet and wait for the beads to pellet.
15. Transfer 50 ul of the supernatant to a new, DNA Lo-Bind tube.

Optional: QC the DNA

1. Measure DNA concentration with 2 ul of the product by absorbance methods. Look for a peak/shoulder at 260nm, if present, proceed to the next step.
2. Assuming sample purity and concentration are adequate, make a 1/10 dilution by diluting 5 uL of the DNA library with 45ul of sterile water. Mix thoroughly and quantify the library on a Bioanalyzer 2100 Hi-Sensitivity chip.
3. If desired, use qPCR to more accurately determine proper loading concentrations of samples prior to sequencing. **Tip:** Make sure that there is no primer-dimer observed on the Bioanalyzer 2100 Hi-sensitivity chip to ensure the qPCR results reflect your desired library fragments and not amplified primers. y
